# Nm23-H1 activator phenylbutenoid dimer exerts cytotoxic effects on metastatic breast cancer cells by inducing mitochondrial dysfunction only under glucose starvation

**DOI:** 10.1038/s41598-021-02729-7

**Published:** 2021-12-07

**Authors:** Bokyung Kim, Jae-Jin Lee, Ji Soo Shin, Ji-Wan Suh, Sunhee Jung, Geum-Sook Hwang, Hee-Yoon Lee, Kong-Joo Lee

**Affiliations:** 1 College of Pharmacy, Graduate School of Pharmaceutical Sciences, Ewha Womans University, Seoul, 03760 Korea; 2 Department of Chemistry, Korea Advanced Institute of Science and Technology, Daejeon, 34051 Korea; 3 Integrated Metabolomics Research Group, Western Seoul Center, Korea Basic Science Institute, Seoul, 03759 Korea

**Keywords:** Cancer, Chemical biology, Drug discovery, Systems biology

## Abstract

Mitochondrial oxidative phosphorylation (OXPHOS) has become an attractive target in anti-cancer studies in recent years. In this study, we found that a small molecule phenylbutenoid dimer NMac1 (Nm23-H1 activator 1), (±)-trans-3-(3,4-dimethoxyphenyl)-4-[(E)-3,4-dimethoxystyryl]cyclohex-1-ene, a previously identified anti-metastatic agent, has novel anti-proliferative effect only under glucose starvation in metastatic breast cancer cells. NMac1 causes significant activation of AMPK by decreasing ATP synthesis, lowers mitochondrial membrane potential (MMP, ΔΨm), and inhibits oxygen consumption rate (OCR) under glucose starvation. These effects of NMac1 are provoked by a consequence of OXPHOS complex I inhibition. Through the structure–activity relationship (SAR) study of NMac1 derivatives, NMac24 was identified as the most effective compound in anti-proliferation. NMac1 and NMac24 effectively suppress cancer cell proliferation in 3D-spheroid in vivo-like models only under glucose starvation. These results suggest that NMac1 and NMac24 have the potential as anti-cancer agents having cytotoxic effects selectively in glucose restricted cells.

## Introduction

Mitochondrial metabolism significantly contributes to cancer proliferation in various ways including ATP production, redox homeostasis, and other metabolic reprogramming. The certain aggressive subtypes of cancer cells were identified to be highly dependent on oxidative phosphorylation (OXPHOS)^1^. Cancer cells also preferentially utilize mitochondrial OXPHOS for energy production in glucose-limiting conditions^2^. Among OXPHOS proteins, complex I is highlighted as an attractive target for high OXPHOS tumors such as pancreatic ductal adenocarcinoma (PDAC), in which the refractory cancers are enriched in complex I at protein and mRNA levels^3^, and invasive breast tumors, in which mitochondrial OXPHOS is favored to produce ATP via enhancement of PGC-1α^4^.

Recent studies have demonstrated OXPHOS inhibitors as anticancer therapeutics targeting the mitochondrial complex I. The biguanides, anti-diabetic drugs for type 2 diabetes, have been identified to reduce carcinogenesis and effectively disrupt cancer cell proliferation by inhibiting complex I^5^. After the reduced cancer risk of metformin-treated type2 diabetes mellitus (T2DM) patients has been reported in 2005, accumulating evidence has supported metformin as an anticancer agent, solely or in combination with other chemotherapy such as paclitaxel^6,7^. The sensitivity of each cell line to metformin treatment is different as 5 mM metformin reduced about 60% of cell viability at 24 h in MDA-MB-231 cells^8^ and IC_50_ values of metformin in 22RV1, PC-3 and LNCaP cells have been identified as 12.3 mM, 2.2 mM and 3.6 mM at 48 h, respectively^6^. Antidiabetic drug thiazolidinediones such as rosiglitazone and pioglitazone have reduced complex I activity as well as uncoupling activity of OXPHOS and both effects lead to mitochondrial impairment^9^. Carboxyamidotriazole (CAI), a calcium channel blocker, is another complex I inhibitor that was effective as an anticancer agent in preclinical studies^10^.

However, the anti-proliferative agents via OXPHOS inhibition are not satisfactory because of low efficacy and high toxicity. As relatively high concentration of metformin is required to inhibit mitochondrial complex 1 of cancer cells, efforts to increase the efficiency of anti-tumorigenic potential yielded phenformin, which is a hydrophobic analogue of metformin as the most potent among the biguanides. However, phenformin has been withdrawn from clinical use because of lethal lactic acidosis, as well as the traditional complex I inhibitor rotenone^11,12^. On the other hand, CAI showed no beneficial effect in patients of non-small cell lung cancer (NSCLC), glioblastoma, and metastatic renal cell carcinoma^13^. Therefore, discovery and development of novel complex I inhibitors having high potency and low toxicity are required for reducing tumorigenesis.

Glucose levels in tumor tissues are up to tenfold lower than normal tissues (5.5 mM) such as 0.1 mM in stomach cancer and 0.4 mM in colon cancer^14^, because of the high rate of glucose consumption with upregulated glycolysis in tumors^15^. Based on the diverse factors including overexpressed levels of glycolytic genes, each cancer type has different sensitivity to this low glucose level. In addition, constant glucose deprived condition is common in solid tumor, because the glucose level is inversely correlated to the distance to capillary bed in many cancer cells^16^. Although cancer cell lines have different sensitivities to low glucose environment^17^, they must adapt their metabolism to the tumor microenvironment. OXPHOS is the major pathway required for optimal proliferation under glucose depleted condition, therefore, tumor cells are sensitive to OXPHOS inhibitors. Most conventional anticancer agents including vincristine, 5-fluorouracil, taxol, doxorubicin, cisplatin, and camptothecin lose their efficacy in glucose deprived tumor environment^18^, while OXPHOS inhibitors can selectively inhibit the glucose-deprived cancer cells. Dual usage of conventional anticancer agent and OXPHOS inhibitor would improve anti-cancer treatment outcome based on their complementary action.

We suggest potential anti-cancer effects of NMac1, Nm23-H1 activator 1, which is a natural product compound cassumunene isolated from *Zingiber cassumunar* Roxb. (Zingiberaceae) and used as an anti-inflammatory agent in East Asia. NMac1 has been reported as having anti-metastatic potential by activating Nm23-H1 having NDP kinase (NDPK) enzymatic activity with direct interaction to C-terminal in vitro and inhibits breast cancer metastasis in vivo^19^.

In this study, we found that NMac1 also has anti-proliferative effects only under glucose starvation in metastatic breast cancer cells. This novel effect of NMac1 is caused by the induction of mitochondrial dysfunction by inhibiting complex 1. We screened NMac1 analogues for anti-proliferative activity under glucose starvation and identified NMac24, through the structure activity relationship (SAR) study, as the most potent agent in anti-proliferation with the same mode of action with NMac1. We further investigated the anti-cancer effect of NMac1 and NMac24 compared with other known complex I inhibitors, biguanides. This study suggests that NMac1 and NMac24 have the potential as anti-cancer agents having cytotoxic effects selectively in glucose restricted condition, in addition to anti-metastatic effect.

## Results

### NMac1 inhibits cancer cell proliferation only under glucose starvation

**Figure 1:**
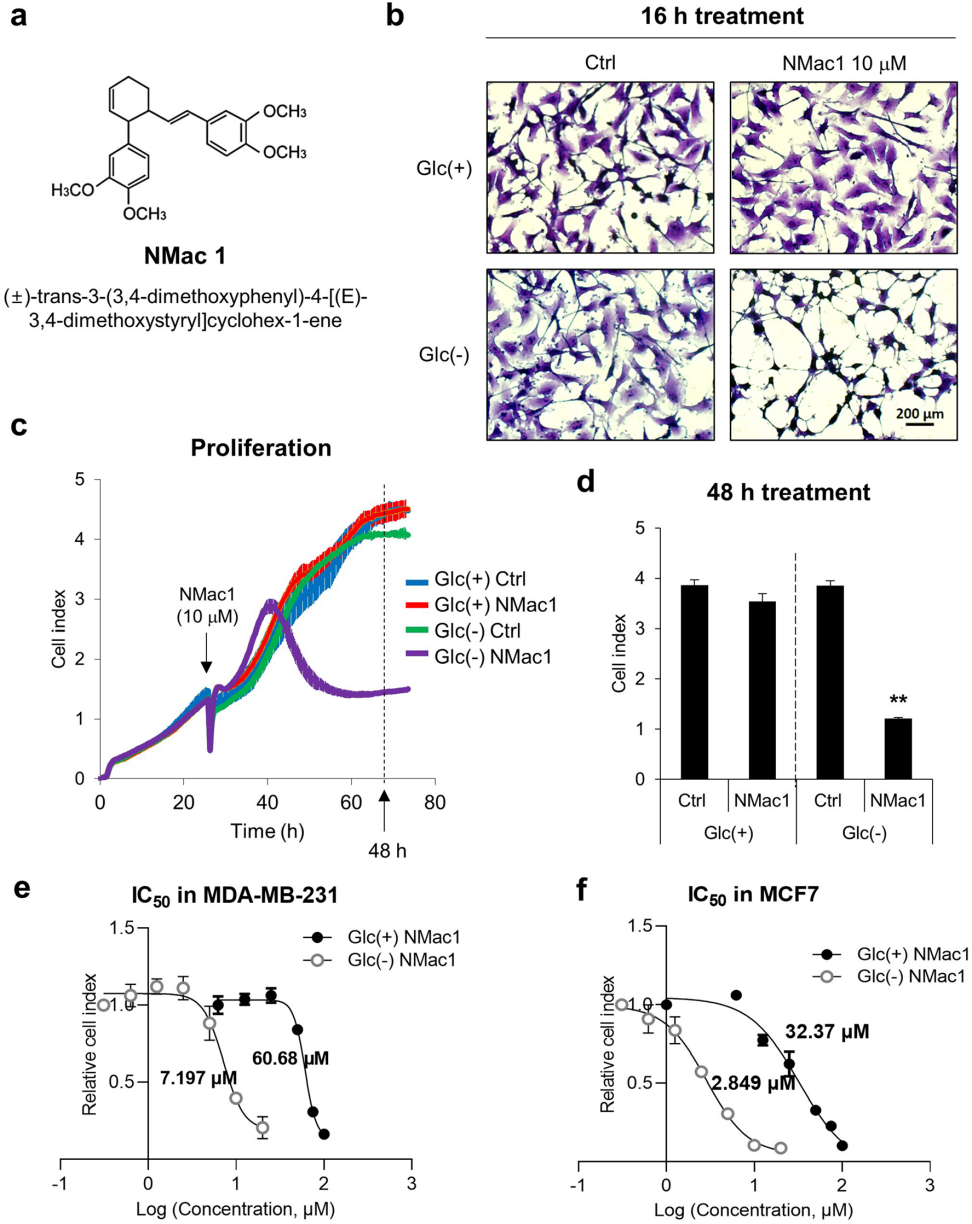
NMac1 induces morphological change and cell death under glucose starvation. (**a**) Chemical structure of NMac1. (**b**) Morphology of MDA-MB-231 cells treated with 10 μM NMac1 or 0.02% DMSO as control with or without glucose for 16 h. (**c**) Real-time cell proliferation and (**d**) cell index at 48 h after NMac1 treatment using xCELLigence RTCA. Glc(+) and Glc(−) represents glucose presence and absence condition, respectively. Error bars represent SD (n = 4). **p < 0.01. IC_50_ values of (**e**) MDA-MB-231 and (**f**) MCF7 cells were calculated with relative cell index at 24 h after NMac1 treatment using Prism 9 (GraphPad software). Error bars represent SD (n = 4).

A small molecule NMac1, (±)-trans-3-(3,4-dimethoxyphenyl)-4-[(E)-3,4-dimethoxystyryl]cyclohex-1-ene (Fig. 1a), is a natural compound isolated from *Zingiber cassumunar* Roxb. (Zingiberaceae)^20^ that is recently identified as an anti-metastatic agent without affecting cell proliferation. To investigate whether NMac1 affects the cell proliferation in tumor microenvironment, we examined the effect of NMac1 on cell proliferation under glucose depleted condition to mimic the tumor microenvironment. MDA-MB-231 cell line was employed as a triple-negative breast cancer (TNBC) model, which has no targeted therapeutics because of triple negative expression in estrogen receptor (ER−), progesterone receptor (PR−) and HER2/neu (HER2−)^21^. MDA-MB-231 cells were treated with NMac1 (10 μM) in the presence and absence of glucose, and the changed morphologies were examined. The substantial morphological changes were observed by NMac1 treatment only under glucose depletion, while no discernible changes were detected by either NMac1 with glucose or glucose starvation without NMac1 (Fig. 1b). To investigate whether the morphological changes are associated with cell proliferation, we monitored the survival of MDA-MB-231 cells in response to NMac1 with or without glucose employing a real-time cell analyzer (RTCA), xCELLigence. Cell survival in response to NMac1 was significantly reduced only under glucose depleted condition (Fig. 1c,d). The results show that NMac1 inhibits the proliferation of MDA-MB-231 cells in the absence of glucose but not in the presence of glucose. The concentration inhibiting 50% of cell proliferation, IC_50_, of NMac1 in MDA-MB-231 cells is 7.20 μM in the absence of glucose, while 60.68 μM in the presence of glucose (Fig. 1e and Supplementary Fig. 1a,b). MDA-MB-231 cells in the absence of glucose are more sensitive to NMac1 than those in the presence of glucose. To ensure that NMac1 inhibits breast cancer cell proliferation in the absence of glucose, we examined the results in another non-invasive breast cancer cell line MCF7. Similar effect of NMac1 was observed, even though the sensitivity is different (Fig. 1f and Supplementary Fig. 1c,d). NMac1 did not affect proliferation of normal cell line MEF in normal glucose condition as shown in Supplementary Fig. 1e,f. To investigate the effect of glucose level on NMac1 action, we examined cell viability in starved (0 mM), low (2.8 mM) and normal (5.5 mM) glucose condition (Supplementary Fig. 1g). NMac1 also slightly reduced cell viability under low glucose condition, suggesting that lower glucose concentration makes cancer cells more sensitive to NMac1. The results demonstrate that NMac1 significantly inhibits the proliferation of cancer cells under glucose starvation, and slightly under low glucose level, mimicking the in vivo microenvironment of solid tumors, while it does not affect their proliferation under the presence of glucose, representing cellular conditions of normal cells.

### NMac1 only in the absence of glucose induces AMPK/mTOR/ERK signaling changes

**Figure 2:**
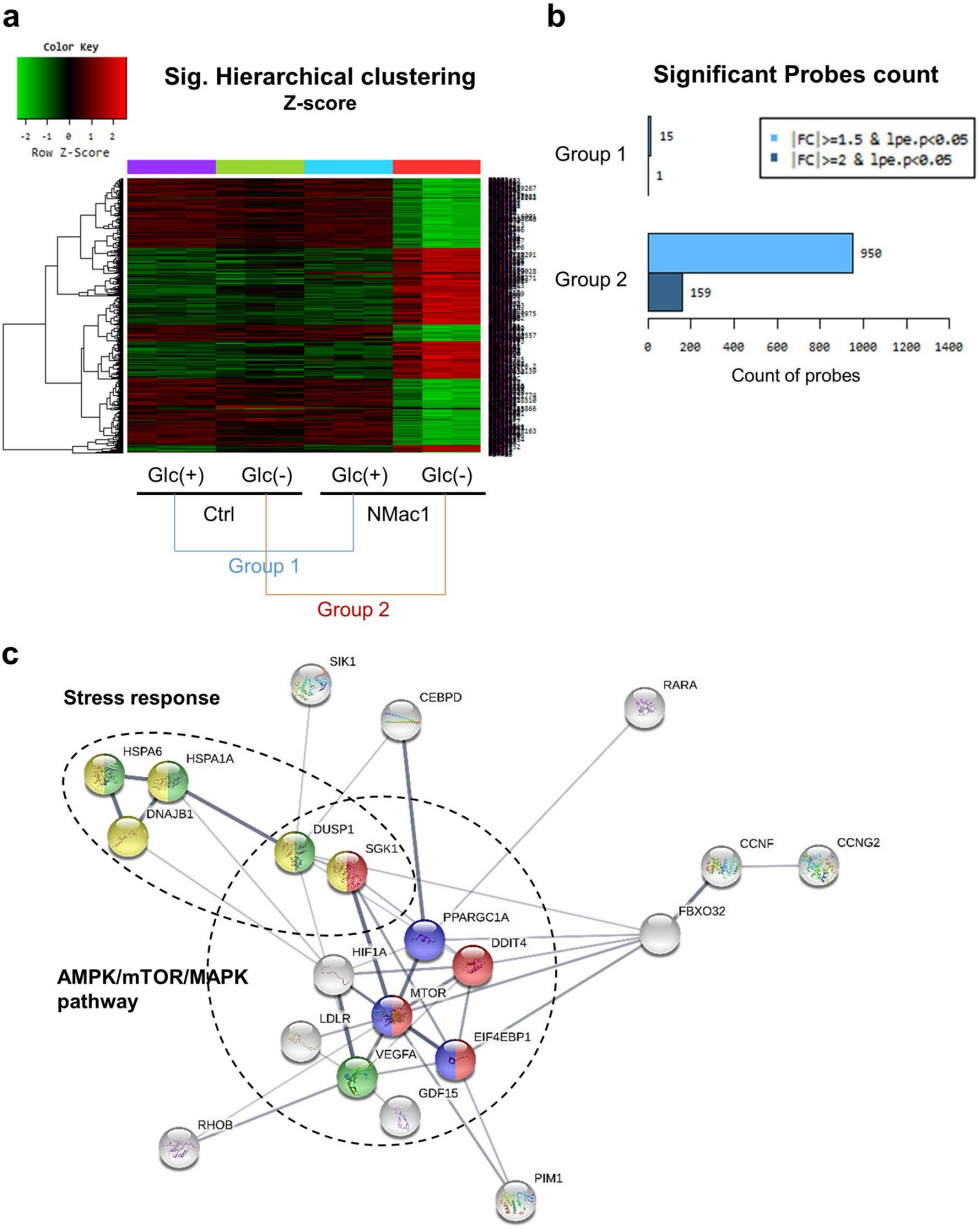
Identification of differentially expressed genes by NMac1 in DNA microarray analysis. (**a**) Significant hierarchical clustering heatmap and (**b**) significant probes count of DNA microarray. (**c**) Significant gene mapping by KEGG pathway analysis was analyzed by STRING based on differentially expressed genes (DEGs) over 2.5-fold changes. The disconnected nodes in the network were hided. Genes colored in yellow, green, blue and red are related to stress response, AMPK pathway, mTOR pathway and MAPK pathway, respectively.

To investigate whether NMac1 induces differential gene expressions in control and glucose depleted condition, we examined global gene expression in MDA-MB-231 cells. To examine the metabolite changes in the live cells, 5 μM NMac1 was treated for 16 h in the absence of glucose and 10 μM NMac1 was treated in the presence of glucose to ensure the metabolite changes by NMac1. The global gene expressions in the NMac1-treated cells were evaluated using DNA microarray analysis. The heatmap shows the patterns of differentially expressed genes (DEGs) that are upregulated (red) or downregulated (green) genes in NMac1-treated cells compared to DMSO-treated control cells. The DEGs by NMac1 treatment compared to control in the presence of glucose were classified as Group 1 and in the absence of glucose were classified as Group 2 (Fig. 2a). Among 47,323 probes, 19,833 genes were filtered by excluding low quality genes (over 50% of replicates in each sample showing *p* value ≥ 0.05 regarded as a low-quality gene).

Fifteen genes in Group 1 and 950 genes in Group 2 were up- or down-regulated based on over 1.5-fold changes with *p* value < 0.05 (Fig. 2b). The DEGs in Group 2 based on 2.5-fold changes and *p* value < 0.05 were analyzed by STRING ver.11.0. Referring to the KEGG (Kyoto Encyclopedia of Genes and Genomes) database^22^, the DEGs in Group 2 were mostly related to AMPK pathway (PPARGC1A and EIF4EBP1, colored in blue), MAPK pathway (HSPA6, HSPA1A, DUSP1 and VEGFA, green), and mTOR signaling pathway (DDIT4, EIF4EBP1 and SGK1, red) (Fig. 2c). AMPK is known as a regulator of mTOR pathway that participates in protein synthesis enhancement under the down-regulator of environmental nutrient changes^23^. The DNA microarray results show the activation of AMPK signaling pathway in response to NMac1 treatment under glucose starvation. In addition, DEGs that are changed over 1.5-fold (data not shown) were also mostly related to AMPK/mTOR/ERK pathway: FBXO32 (2.64-fold upregulated), known to be inversely regulated with mTOR for protein synthesis; RUNX1 (2.32-fold downregulated) and BDNF (1.81-fold upregulated), regulated by ERK and contribute to cell migration, invasion and growth^24,25^; EGFR (1.79-fold upregulated), identified to increase cell growth metabolism via MAPK/ERK pathway and cell survival via mTOR pathway^26^. The results show that NMac1 treatment without glucose mainly regulates AMPK/mTOR/ERK pathway in MDA-MB-231 cells. Another DEGs including HSPA6, HSPA1A, DNAJB1, DUSP1, DDIT3 and SGK1 (colored in yellow), were identified to be changed in response to various stresses. HSP70^27^, GDF15^28^, FBXO32^29^ and IL6^3^, which were upregulated in gene expression level, are related to hypoxic stress similar to glucose deprivation and heat shock. Those are also known as essential genes in cancer proliferation or epithelial-to-mesenchymal transition (EMT) under hypoxia and related to hypoxia-induced autophagy^29^. To investigate whether NMac1 induces autophagic cell death, we examined the protein expression level of Hsp70 and 4EBP1, which was the most upregulated in gene expression level, PARP degradation and activation of caspase 3 and LC3 related to apoptosis and autophagy. No discernible changes in the expression of Hsp70 and 4EBP1 were observed, but p-4EBP1, a direct target of mTOR, is significantly decreased, which contributes to protein translation^30^ (Supplementary Fig. 2a,b). Also, we found that the NMac1-induced cell death is not occurred from autophagy nor apoptosis because no discernible changes in PARP and caspase 3 as apoptotic cell death markers, and LC3 as an autophagic cell death marker are observed (Supplementary Fig. 2c). Further studies are needed to understand the NMac1-induced cell death other than apoptosis or autophagy.

**Figure 3:**
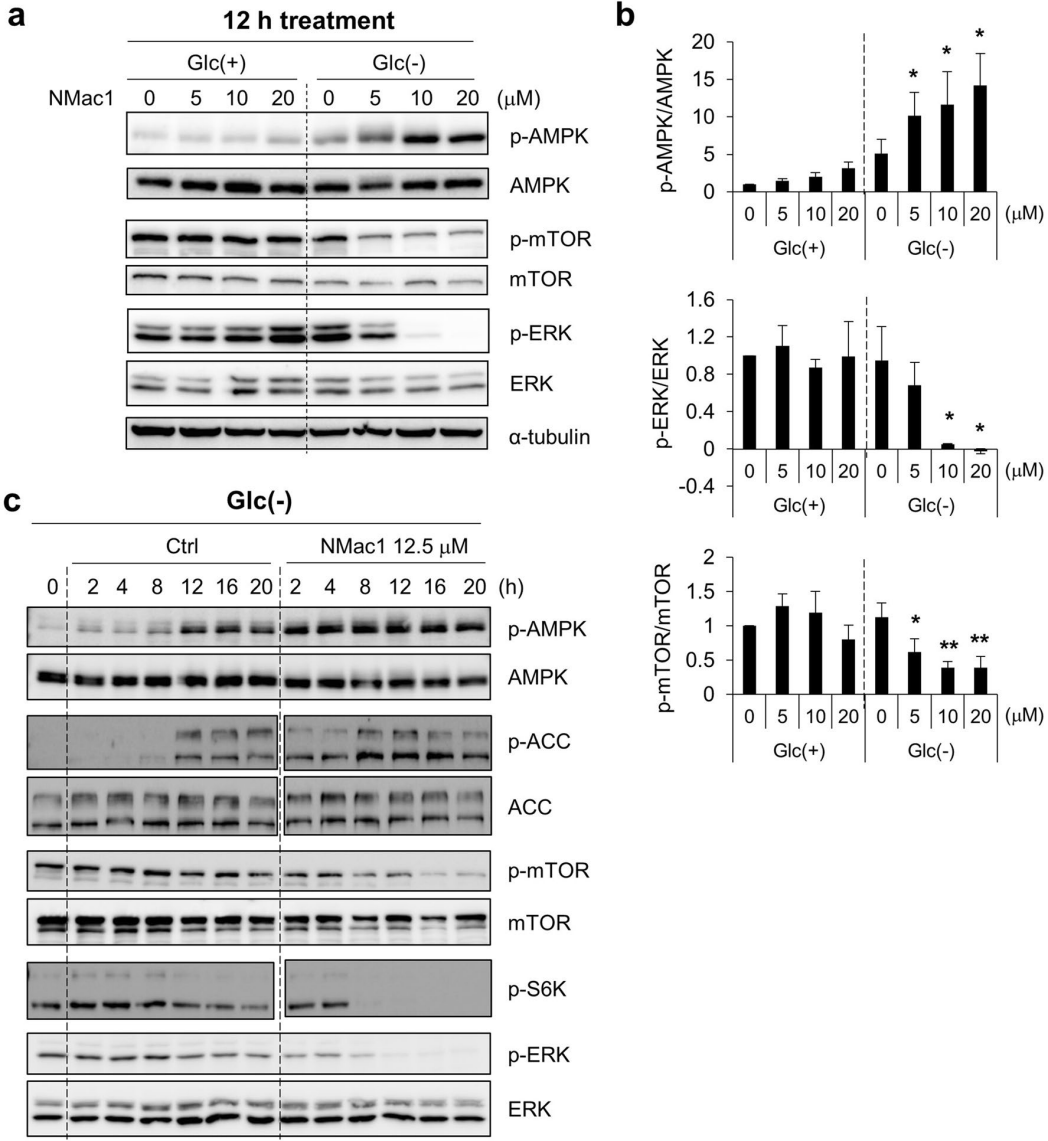
NMac1 treatment in the absence of glucose induces AMPK activation and inhibits mTOR and ERK. (**a**) Representative images of western blot analysis for AMPK/mTOR/ERK signaling changes and (**b**) quantified relative expressions using Multigauge ver.3.0 in MDA-MB-231 cells. Indicated concentrations of NMac1 were treated for 12 h under the presence and absence of glucose. Error bars represent SD (n = 3). *p < 0.05, **p < 0.01. (**c**) Kinetics of AMPK/mTOR/ERK signaling changes by western blot analysis. MDA-MB-231 cells were treated with 12.5 μM NMac1 under the indicated condition. Full-length blots are presented in Supplementary Fig. 7.

To ensure that NMac1 induces metabolic stresses, we examined the AMPK activation as well as ERK and mTOR signaling. MDA-MB-231 cells were exposed to various concentrations of NMac1 in the presence and absence of glucose. NMac1 treatment only under glucose starvation induced AMPK activation and mTOR/ERK inhibition in a concentration-dependent manner, while only one of either glucose depletion or NMac1 treatment does not activate AMPK (Fig. 3a,b). To confirm the AMPK activation under glucose starvation, we examined the kinetics of AMPK activation as well as p-mTOR, p-ERK, p-S6K (glucose-induced ribosomal protein S6 kinase) in response to 12.5 μM NMac1 in MDA-MB-231 cells. We found that NMac1 under glucose depletion rapidly induced AMPK activation in 2 h, while glucose starvation without NMac1 in control took more than 8 h (Fig. 3c). This AMPK activation kinetics are well agreed with those of mTOR, ERK and S6K inactivation. These results indicate that NMac1 induces the activation of AMPK only in glucose depleted condition.

### NMac1 induces mitochondrial dysfunction by inhibiting ATP production and reducing the mitochondrial membrane potential

**Figure 4:**
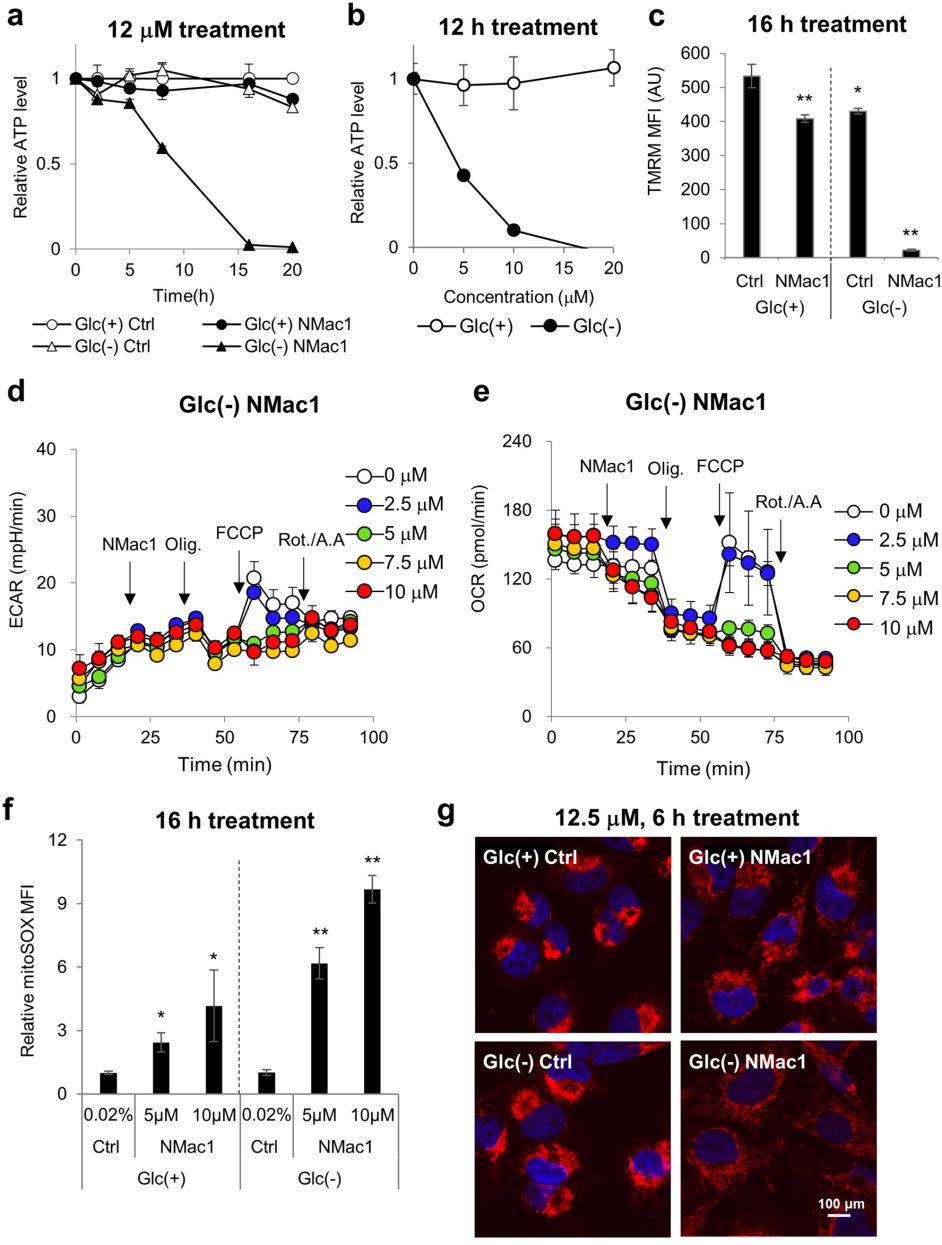
NMac1 induces ATP depletion in a concentration dependent manner via mitochondrial dysfunction. (**a**) Time-dependent cellular ATP level in MDA-MB-231 cells. 12 μM NMac1 or 0.025% DMSO were treated for indicated time. (**b**) Cellular ATP levels were examined after 12 h of indicated concentrations of NMac1. (**c**) Mitochondrial membrane potential (MMP) with NMac1 treatment in MDA-MB-231 cells. 10 μM NMac1 was treated with or without glucose for 16 h. (**d**) ECAR and (**e**) OCR of MDA-MB-231 cells were monitored with Seahorse XF96 analyzer (Agilent Technologies Inc.). NMac1 (0, 2.5, 5, 7.5 and 10 μM), oligomycin (1 μM), FCCP (0.5 μM) and rotenone/antimycin A (0.5 μM) were injected sequentially at each time point. Error bars represent SD (n = 4). (**f**) Mitochondrial ROS level was measured in MDA-MB-231 cells treated with 0.02% DMSO, 5 or 10 μM NMac1 using FACS Calibur (BD Biosciences). Error bars represent SD (n = 3). *p < 0.05, **p < 0.01. (**g**) Fluorescence microscopic images of mitochondrial morphology. MDA-MB-231 cells were treated 12.5 μM NMac1 for 6 h and stained with TOM20 (red, mitochondria) and DAPI (blue, nucleus).

AMPK is known to be activated by increasing cellular AMP/ATP or ADP/ATP ratios and decreasing cellar energy level. To investigate whether NMac1 regulates cellular ATP level, we examined the kinetics of intracellular ATP level in MDA-MB-231 cells. Cells were incubated with control or 12 μM NMac1 with or without glucose. NMac1 significantly depleted the cellular ATP level only when glucose is depleted in a time-dependent manner, while glucose depletion itself without NMac1 did not affect cellular ATP level (Fig. 4a). The ATP depletion was also confirmed by treating cells with various concentrations of NMac1 in the presence and absence of glucose for 12 h. As shown in Fig. 4b, ATP levels were decreased by NMac1 in the absence of glucose in a concentration dependent manner. ATP depletions are well agreed with the cell survival (Fig. 1) and AMPK/mTOR/ERK signaling changes (Fig. 3). The results suggest that NMac1-induced cell death under glucose starvation is caused by the rapid cellular ATP depletion.

To investigate whether NMac1-dependent ATP depletion under glucose starvation is associated with the inhibition of the electron transport chain (ETC), we examined the mitochondrial membrane potential (MMP, ΔΨm) in response to NMac1 treatment with and without glucose. The MMP generated by proton pumps (Complexes I, III and IV) forms the transmembrane potential of hydrogen ions, which are utilized to generate ATP. MMP of MDA-MB-231 cells treated with 10 μM NMac1 for 16 h with and without glucose was measured using TMRM fluorescent dye-based flow cytometry. Among the samples, MMP of cells treated with NMac1 under glucose starvation was significantly reduced, while no discernible differences were observed in cells treated with NMac1 in the presence of glucose or with control in the absence of glucose (Fig. 4c). The results of MMP are well corresponded with NMac1 caused-ATP depletion in cells. In summary, NMac1 in the absence of glucose inhibits mitochondrial ETC, reduces proton pumping that might depolarize the mitochondrial membrane, and inhibits ATP synthesis.

### NMac1 induces ATP depletion by reducing oxygen consumption rate and producing mitochondrial ROS

To determine how NMac1 induces ATP depletion, we examined the function of ATP generation processes including glycolysis and oxidative phosphorylation (OXPHOS) in MDA-MB-231 cells in response to 10 μM NMac1 treatment in the absence of glucose. The glycolysis was assessed by measuring extracellular acidification rate (ECAR) and OXPHOS was assessed by measuring oxygen consumption rate (OCR) employing Seahorse XF analyzer (Agilent). Modulators of cellular respiration that specifically target components of the electron transport chain (ETC) were serially injected as indicated for measuring key parameters of metabolic function. The compounds, oligomycin (ATP synthase inhibitor), FCCP (uncoupler) and a mixture of rotenone/antimycin A (Complex I and III inhibitor, respectively), contribute to detect basal ATP-linked respiration, maximal respiration, and non-mitochondrial respiration, respectively. While cellular ECAR was not affected (Fig. 4d), OCR was significantly inhibited immediately after NMac1 treatment in a concentration-dependent manner (Fig. 4e). This suggests that NMac1 induces dysregulation of mitochondrial ATP production system OXPHOS followed by total depletion of cellular ATP level.

Mitochondrial ROS (mtROS) are easily generated from electron leakage at mitochondrial complex I and III, and quickly converted to H_2_O_2_ by superoxide dismutase (SOD)^31^. To investigate whether ETC inhibition by NMac1 causes rapid ROS elevation in mitochondria, we examined mitochondrial ROS, mainly superoxide. MDA-MB-231 cells were treated with 5 or 10 μM NMac1 for 16 h and mtROS were measured by MitoSOX-based flow cytometry. The mtROS level was significantly increased by NMac1 treatment in the absence of glucose, while relatively weak increase in presence of glucose (Fig. 4f). The substantial increases of mtROS by NMac1 treatment in the absence of glucose were confirmed in a time-dependent manner, while slightly increased mtROS in the presence of glucose at 2 h were maintained afterwards (Supplementary Fig. 3a). The excess production of mtROS is associated with mitochondrial dysfunction, resulting the increase of mitochondrial mass & mitochondrial DNA (mtDNA) fragmentation, alteration of mitochondrial respiratory complexes, cytochrome *c* (Cyt *c*) release, and decrease of MMP and mitochondrial transcription factor A (TFAM)^32^. To ensure whether the increase of mtROS by NMac1 is associated with mitochondrial function, we examined the mitochondrial morphology in response to NMac1 treatment with and without glucose. MDA-MB-231 cells were treated with 12.5 μM NMac1 in the presence and absence of glucose for 6 h, and the morphology of mitochondria was detected by Tom20 staining. NMac1 in the absence of glucose deteriorates the mitochondrial integrity of cells (Fig. 4g).

To investigate whether mtROS elevation is the main cause of NMac1 induced cytotoxic effect, we examined the morphological changes in response to NMac1 by scavenging the mtROS using mitoTEMPO. As shown in Supplementary Fig. 3b, reducing the mtROS with mitoTEMPO does not affect anti-proliferation induced by NMac1. The results suggest that increase of mtROS is not a main cause of cell death, rather it is generated by the results of NMac1 induced mitochondrial dysfunction, including loss of MMP and decrease of oxygen consumption rate and ATP depletion.

### Metabolomic profiling results confirm the mitochondrial dysfunction, induced in NMac1-treated MDA-MB 231 cells under glucose depleted condition

**Figure 5:**
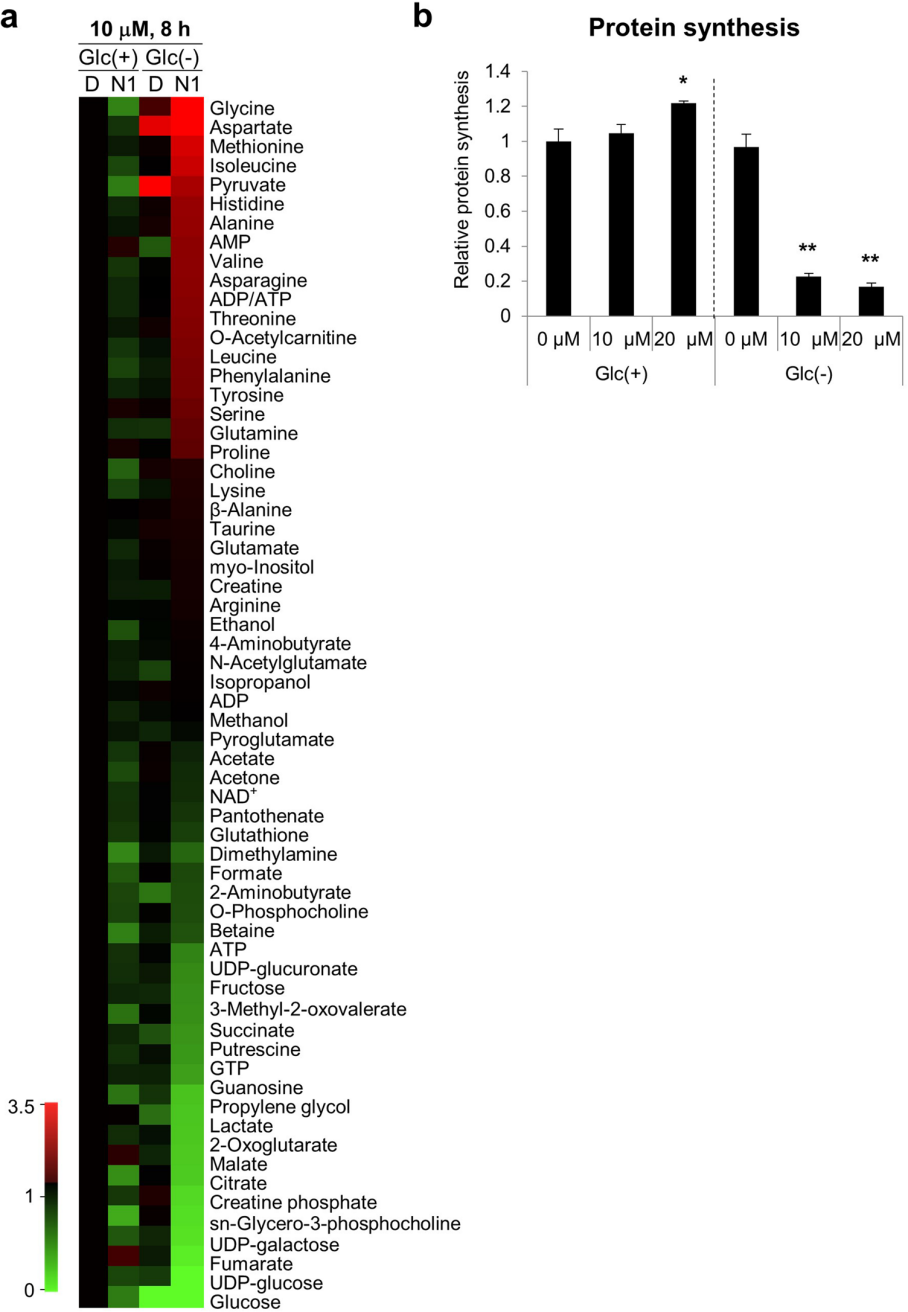
Metabolomics profiles of MDA-MB-231 cells treated with NMac1. (**a**) Heatmap of metabolomics analysis in MDA-MB-231 cells. Cells were treated with 10 μM NMac1 with and without glucose for 8 h. Green and red color represent down- or up-regulated level based on fold changes compared to each metabolite level of Glc(+) DMSO. (**b**) Quantification of protein synthesis analyzed using puromycin assay. MDA-MB-231 cells were treated 0.025% DMSO (Ctrl), 10 or 20 μM NMac1 with and without glucose for 8 h. Puromycin (0.5 μg/mL) was added and incubated for last 2 h. Puromycin incorporation by protein synthesis was detected by western blot analysis. Error bars represent SD (n = 3). *p < 0.05, **p < 0.01.

In order to investigate whether NMac1 affects the mitochondria-associated metabolic changes specifically under glucose starvation, we examined the metabolic changes in MDA-MB-231 cells treated with NMac1 in the presence and absence of glucose, employing NMR-based metabolomics analysis. Cells were treated with 10 μM NMac1 or 0.02% DMSO as a control for 8 h, when ATP levels are steeply decreased in the absence of glucose. Metabolites of each group were measured triplicated, or the reproducibility of metabolite changes of each group was measured in 5 samples (Fig. 5a). The separation of 4 groups for metabolite profiles was confirmed by multivariate unsupervised principal component analysis (PCA). As the groups are separated along the projected plane, they are expected to have different profiles by each treated condition, which represents that samples in different groups can be explained as belonging to different groups (Supplementary Fig. 4a). Metabolomics experimental setup is well consistent with above results in terms of glucose concentration and ADP/ATP ratio (Supplementary Fig. 4b). NMac1 in the absence of glucose inhibits TCA cycle, protein synthesis and gluconeogenesis (Supplementary Fig. 4c). Inhibition of TCA cycle by NMac1 is resulting in the decrease of intermediates in TCA cycle, including succinate, 2-oxoglutarate, malate, citrate, and fumarate. Considerable accumulation of most amino acids by NMac1 is possibly caused by the prevention of protein synthesis. Suppression of protein synthesis by NMac1 was examined employing puromycin-based protein synthesis assay and we found that NMac1 clearly inhibits the protein synthesis in the absence of glucose (Fig. 5b and Supplementary Fig. 4d). NMac1 without glucose impedes the gluconeogenesis by decreased gluconeogenic precursors including 2-oxoglutarate and NAD^+^, which induces the increases of glucogenic precursors: pyruvate and Asp. Also, NMac1 treatment reduces another group of gluconeogenenic precursors from glycogen degradation, UDP-glucose, UDP-galactose and UDP-glucuronate that are metabolized to glucose-1-phosphate. Inhibition of gluconeogenesis by NMac1 abrogates the compensatory process in glucose deprived condition and induces cell death. It is known that the gluconeogenesis pathway becomes vital when the exogenous or endogenous glucose supply is insufficient^33^.

Gluconeogenesis is the major energy producing pathway to compensate for the energy shortage during fasting via utilizing intra- or extra-cellular sources such as amino acids^34^. Many cancer cells in prolonged fasting or glucose deprivation condition are more likely to show abbreviated forms of gluconeogenesis, which provides lactate, Gln and several other amino acids to biosynthetic pathways^35^. The decreased lactate and Gln levels that are important non-carbohydrate sources in cancer^36^, were apparent in our data. As cancer cells are starved in nutrients, especially glucose, by high demand and incomplete angiogenesis^37^, they go under metabolic reprogramming and thus, the related metabolites are consumed faster and exist in lower levels under glucose starvation (Fig. 5a).

However, cells maintain normal growth and ATP level under glucose starvation alone (Figs. 1 and 4a) despite the inhibited patterns in gluconeogenesis (Fig. 5a and Supplementary Fig. 4c). This indicates that ATP supply is compensated by various other pathways. In addition to the decrease of gluconeogenesis intermediates, glucose starved cells after NMac1 treatment show the decrease of metabolites in TCA cycle, protein synthesis, energy source ATP and creatine phosphate, and increase most of amino acids. The results suggest that NMac1 makes the cells more sensitive and lethal to glucose starved condition, although further studies are required for understanding the molecular mechanism of NMac1 acting on these processes.

### NMac1 inhibits mitochondrial complex I activity

Various cell lines have different sensitivities to low-glucose condition and the sensitivity is decided by the capability of OXPHOS upregulation. The sensitive cell line to low-glucose condition has defect in OXPHOS upregulation in response to glucose depleted condition^17^ and the defects are mainly caused by either mitochondrial DNA (mtDNA) mutations in complex I genes or impaired glucose utilization. The resistant cell lines to glucose depletion are sensitive to OXPHOS inhibitors, especially to complex I inhibitors^2^.

**Figure 6:**
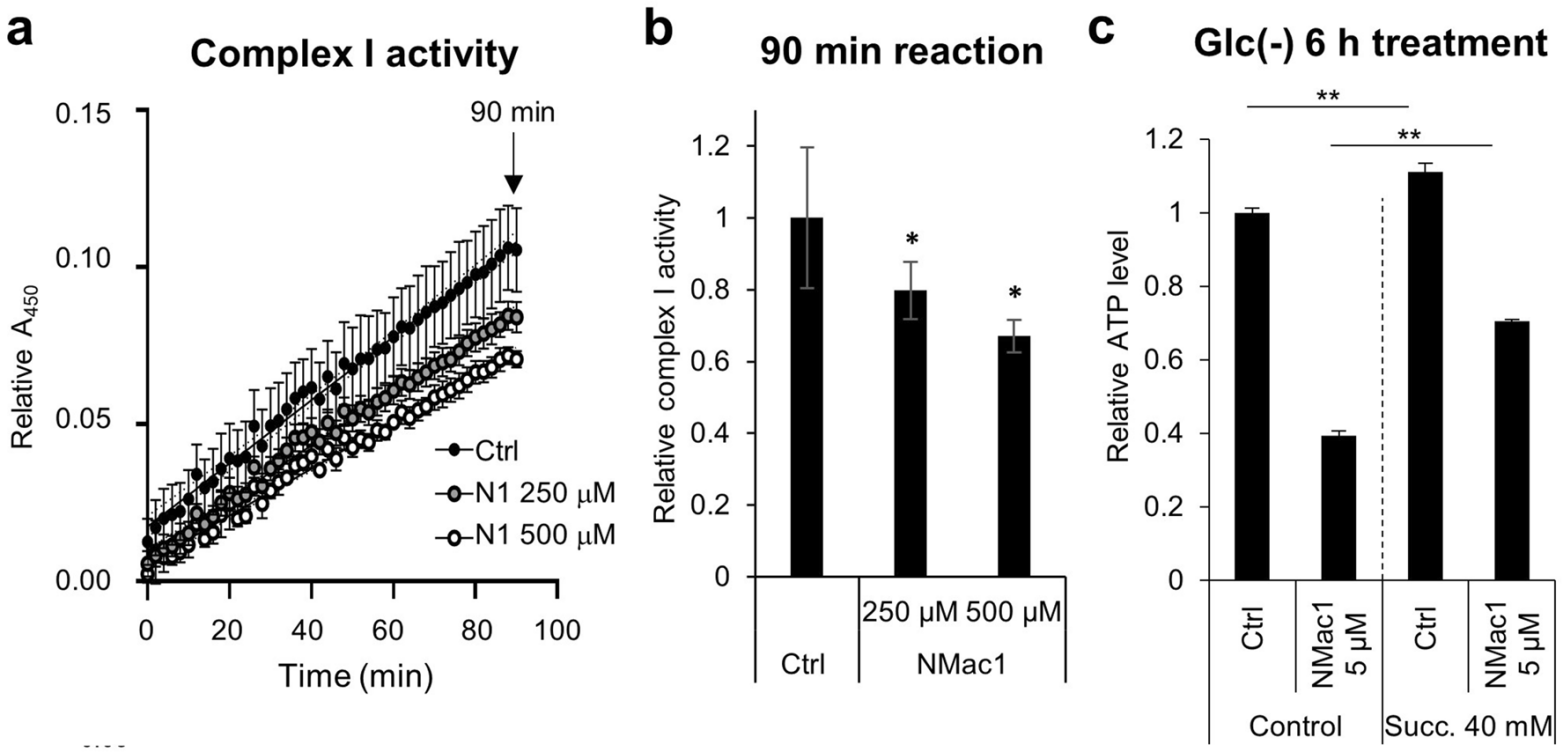
NMac1 inhibits mitochondrial complex I enzyme activity. (**a**) Kinetics and (**b**) relative activity at 1.5 h of complex I in MDA-MB-231 cells. N1 represents NMac1. (**c**) Relative ATP level was measured by 0.01% DMSO (control) or 5 μM NMac1 treatment with or without 40 mM dimethyl-succinate addition. Error bars represent SD (n = 3). *p < 0.05, **p < 0.01.

MDA-MB-231 cells are resistant to low-glucose condition. In addition, MMP is established by proton pumps in complexes I, III and IV proton pumps and is an essential component in ATP synthesis in mitochondria^38^. Specifically, mitochondrial complex I, a NADH-ubiquinone oxidoreductase, mediates majority of electron transfer for ATP generation through complex III, an ubiquinol-cytochrome *c* oxidoreductase. Complex I and III are known to generate most of superoxide in mitochondria^39^ by electron leakage, therefore, inhibition of those complexes might cause the rapid elevation of mitochondrial ROS because of more electron leakages, and the decrease of MMP. Since NMac1 significantly deteriorated MMP, induced ATP depletion and inhibited mitochondrial OCR, it is possible that NMac1 inhibits either complex I or complex III. To investigate whether NMac1 induces ATP depletion via collapse of MMP by inhibiting mitochondrial complex I, we examined enzymatic activity of complex I in MDA-MB-231 cell lysates obtained from cells treated with various concentrations of NMac1 in the absence of glucose. NMac1 reduces complex I activity in a concentration-dependent manner (Fig. 6a, b). To ensure that the NMac1-caused ATP depletion is a direct inhibition effect of NMac1 on complex I, we examined ATP levels in cells adding the dimethyl succinate, the membrane permeable substrate of complex II, after NMac1 treatment. When complex I-mediated respiratory activity is inhibited by inhibitors such as rotenone, the addition of dimethyl succinate is known to be transported into mitochondria through the dicarboxylate carrier and oxidized to fumarate with the reduction of FAD to FADH_2_^40^. Addition of dimethyl succinate in vitro was identified to ameliorate about 20% of OCR rate and 2,3-^13^C_2_ succinate (disodium salt) administration in vivo by cerebral micro-dialysis in traumatic brain injury (TBI) patients improved mitochondrial function^41,42^. The decreased ATP level by NMac1 without glucose was partly compensated by adding dimethyl succinate (Fig. 6c), indicating that NMac1 inhibits complex I, not complex III. The results demonstrate that NMac1 induces ATP depletion by inhibiting mitochondrial function through the inhibition of complex I activity.

### Novel Nm23/NDPK activator analogues are found based on structure–activity relationship (SAR) study

Based on backbone structure of NMac1, novel NMac1 analogue compounds were designed, synthesized and screened their activities for anti-metastasis by measuring NDPK activity (data not shown) and anti-proliferation by staining the nuclei of adherent cells with crystal violet (CV) (Supplementary Fig. 5a). NMac1 was previously identified to activate the NDPK activity of Nm23-H1 and increased the anti-metastatic potential. The activation of NDPK by NMac1 significantly reduced cell invasion in vitro via Rac1 inhibition and cancer metastasis in vivo^19^, and thus NDPK activity increased by NMacs is positively correlated with anti-metastatic potential of the compounds. The activities of NMac1 analogues in anti-metastasis and anti-proliferation from each screening method were summarized in Supplementary Table 2. The SAR study of NMac1 analogues based on this screening revealed that four methoxy groups are required for both activities (anti-metastatic activity by activating NDPK activity and anti-proliferative activity). Two distinct structural series were identified. One series (NMac1, 21, 24, 25) allows spatial flexibility of the two catechol units to accommodate both anti-proliferative and anti-metastatic activities. The other series (NMac22, 23, 28–31, 33, 38) keeps the two catechol units in the same plane to allow only anti-metastatic activity by activating NDPK activity, not anti-proliferative activity.

The anti-proliferative effect, which affected cell viability in CV staining, was further investigated to compare the effectiveness in cell proliferation. NMac24 was identified as the most potent compound in anti-proliferation with IC_50_ = 2.46 μM (Supplementary Fig. 5b and 6e) and NMac38 is the most potent in anti-metastatic activity, but no anti-proliferative effect. In summary, NMac1 shows both the anti-metastatic activity as activating Nm23-H1/NDPK-A and the anti-proliferative activity by inducing ATP depletion and mitochondrial dysfunction in the absence of glucose. The SAR study revealed that these two activities of NMac1 arose from activation/inhibition of two distinct systems as NMac38 showed only the anti-metastatic activity. Throughout SAR study, novel compound NMac24 was identified as a representative compound in anti-proliferation and NMac38 as a representative compound in anti-metastasis without anti-proliferative activity.

**Figure 7:**
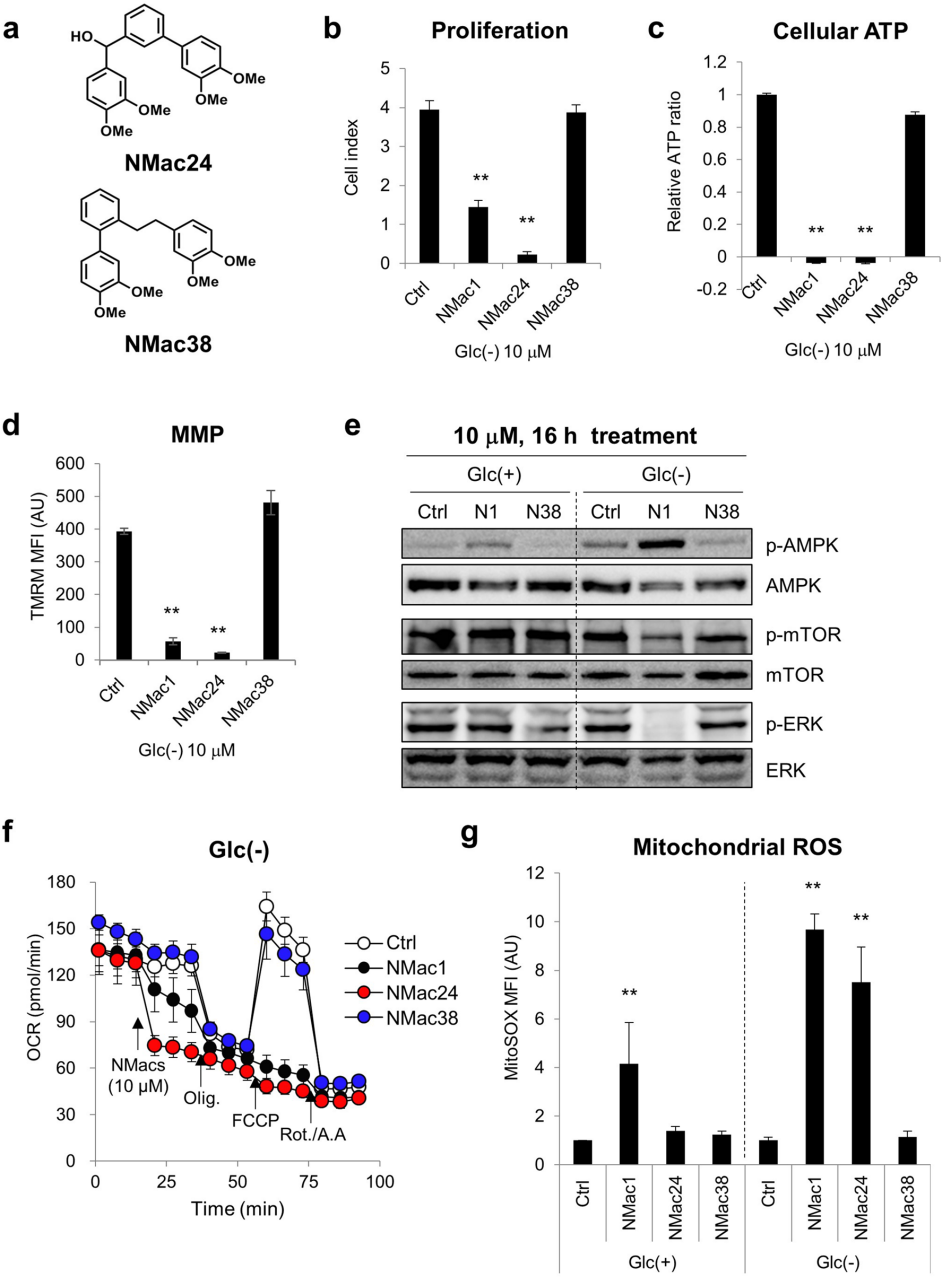
Comparison of mitochondrial dysfunction between NMac1 analogues. (**a**) Structures of NMac24 and NMac38. (**b**) Cell proliferation of MDA-MB-231 cells measured with xCELLigence RTCA after 10 μM of each chemical treatment for 48 h under glucose starvation. (**c**) Cellular ATP levels, (**d**) mitochondrial membrane potential (MMP) and (**e**) AMPK/mTOR/ERK signaling by western blot analysis were examined after 10 μM of each chemical treatment for 16 h under glucose presence or starvation, presented as Glc(+) or Glc(−). N1 and N38 represents NMac1 and NMac38. (**f**) OCR of MDA-MB-231 cells was measured with Seahorse XF96 analyzer (Agilent Technologies Inc.). Chemicals (DMSO (ctrl), NMac1, NMac24 or NMac38), oligomycin (1 μM), FCCP (0.5 μM) and rotenone/antimycin A (0.5 μM) were injected sequentially at each time point. (**g**) Mitochondrial ROS level. Cells were treated 0.02% DMSO (Ctrl) or 10 μM chemicals. Error bars represent SD (n = 3). *p < 0.05, **p < 0.01. Full-length blots are presented in Supplementary Fig. 7.

To investigate whether anti-proliferative activity is distinguished from the mechanism of anti-metastasis, the NMacs including NMac1, NMac24 and NMac38, were examined the anti-proliferative potential by cell proliferation, cellular ATP level, MMP, OCR and AMPK/mTOR/ERK signaling in MDA-MB-231 cells. NMac24 significantly inhibits cancer cell proliferation, induces ATP depletion, decreases MMP and OCR, causes AMPK/mTOR/ERK signaling changes in addition to the dramatic mtROS elevation that were consistent results with NMac1 (Fig. 7 and Supplementary Fig. 6). NMac24 suggests higher potency than NMac1 in all markers of mitochondrial dysfunction (Fig. 7d,f and Supplementary Fig. 6a,c,d,f). On the other hand, NMac38, negative control in anti-proliferation, shows no discernible effects on inducing mitochondrial dysfunction in various factors (Fig. 7b–g, Supplementary Fig. 6b,g).

The inhibition of oxygen consumption rate by NMac1 and NMac24 but not by NMac38 is also consistent with the above results (Supplementary Fig. 6f,g). Although additional activities of mitochondrial complexes are still needed to be identified, over 5 μM of NMac1 and NMac24 induced total decrease of OCR (Fig. 4e and Supplementary Fig. 6f), which suggests that NMac1 and NMac24 possibly inhibit complex I or III when inducing mitochondrial dysfunction. In addition, the ATP decrease by NMac1 was compensated as succinate addition (Fig. 6c), which supports the complex I inhibition instead of complex III.

The results demonstrate that NMac1 and NMac24 have anti-proliferative activities by inducing mitochondrial dysfunction under glucose starvation, and these activities are separated from anti-metastatic activities by activating metastasis suppressor NDPK.

### Anti-tumor activities of NMac1 and NMac24 are confirmed using in vivo-like 3D spheroid cancer model

To investigate whether NMac1 and NMac24 have anticancer activity in vivo, we examined the effect of the compounds employing in vivo-like 3D spheroid cancer model. Although monolayer cell culture models are still widely used because of its high reproducibility and easy manipulation, they have limitations to reproduce cancer microenvironment because of monolayer and manipulated conditions. 3D spheroid models, on the other hand, show more precise effects of chemical compounds via presenting cancer microenvironment^43^ including up-regulated EMT-associated proteins^44^ and pharmacokinetic obstacles, which limit actual drug penetration to core part of cancer tissues and have hypoxic, nutrient-deficient, and glucose deficient condition of this part^15,45^. Therefore, 3D-spheroid models are employed for screening of novel anticancer therapeutics and widely utilized as intermediary procedure between in vitro and in vivo models^46^.

**Figure 8:**
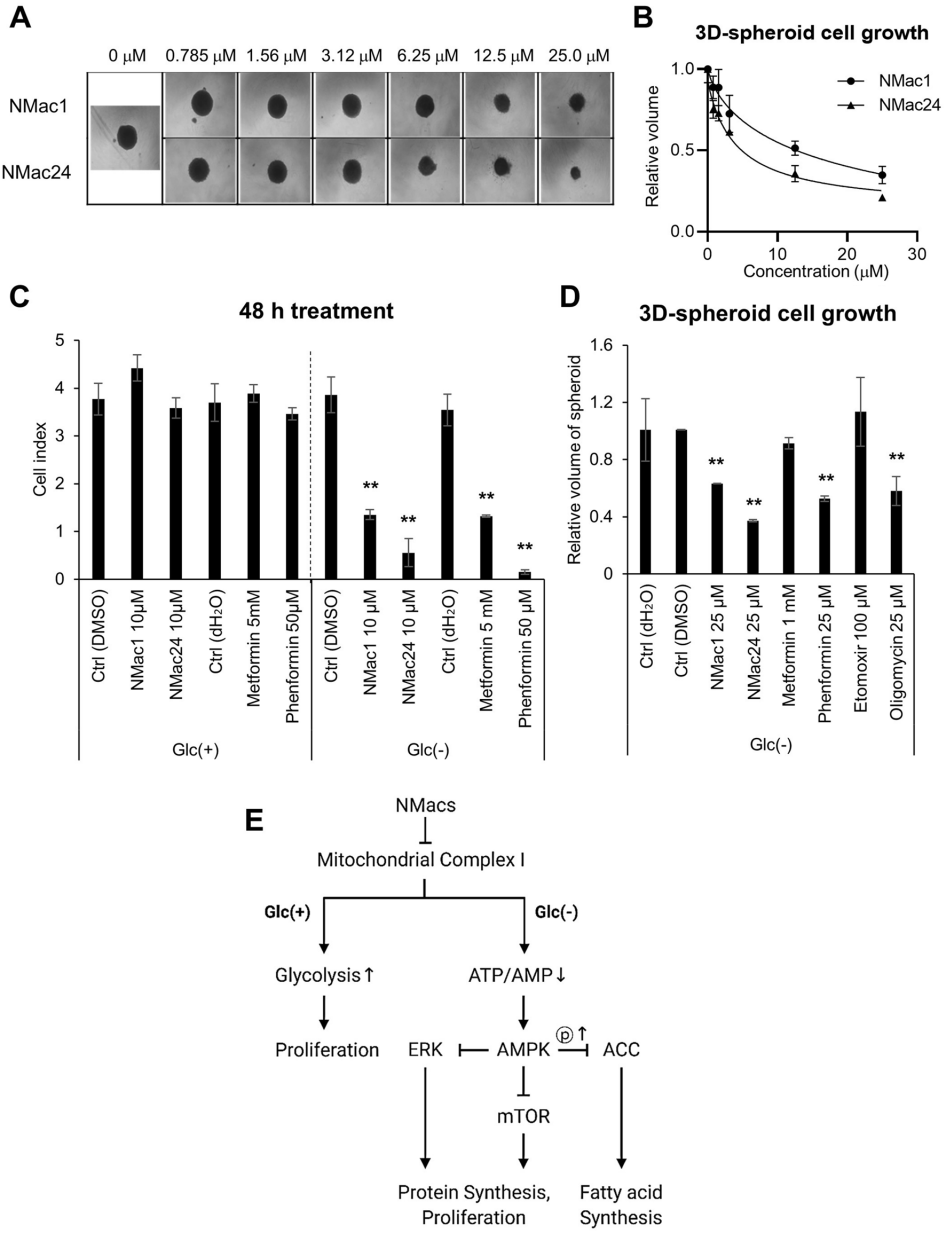
Effect of NMac1 and NMac24 on 3D spheroid cell proliferation. (**a**) Representative images and (**b**) relative volume of three-dimensional (3D) spheroid cell proliferation. MDA-MB-231 cells were seeded in 2.5% matrigel, then after initiation, NMac1 and NMac24 were treated. Each spheroid size was measured after 2 weeks of chemical treatment. Error bars represent SD (n = 3). (**c**) Cell index using xCELLigence RTCA and (**d**) relative volume of 3D spheroids were measured after indicated concentrations of chemical treatment under glucose starvation for 48 h and 2 weeks, respectively. Error bars represent SD (n = 3). *p < 0.05, **p < 0.01. (**e**) Scheme of NMacs’ action on anti-proliferation. NMacs represent NMac1 and NMac24.

To confirm the anticancer activities of NMac1 and NMac24, we examined the volume of each spheroid obtained from MDA-MB-231 spheroid cells which were treated with NMac1 or NMac24. Treatment of the compounds under glucose starvation inhibits the proliferation of MDA-MB-231 spheroid cells in a dose-dependent manner (Fig. 8a,b). NMac24 shows more substantial inhibition of tumor cell growth compared to NMac1. The results suggest that the NMac1 and NMac24 have therapeutic potential as anti-cancer agents in metastatic breast cancer model.

### NMac1 and NMac24 have higher anti-proliferative potential than other known complex I inhibitors

Since NMac1 and NMac24 show anticancer activities via inhibiting mitochondrial complex I, we compared the anti-proliferative activities of NMac1 and NMac24 with other known mitochondrial complex I inhibitors. Metformin and phenformin, which originally have been used as T2DM, have identified their anticancer potential via complex I inhibition in recent studies^5,47^. Etomoxir, an irreversible inhibitor of carnitine palmitoyl transferase 1 (CPT-1) as a mitochondrial enzyme involved in fatty acid β-oxidation, is also identified to inhibit complex I of the electron transport chain by an off-target effect at concentrations above 10 μM^48,49^. MDA-MB-231 cells were treated with different concentration of each compound in the presence and absence of glucose by using the minimum effective concentration by optimization: 10 μM NMac1 and NMac24, 50 μM phenformin, and 5 mM metformin. The cell proliferation was measured using xCELLigence. Phenformin (50 μM) shows the most effective anti-proliferative activity after 48 h of chemical treatment, then followed by 10 μM NMac24/NMac1 and 5 mM metformin (Fig. 8c). The anti-cancer effects were further confirmed employing 3D spheroid in vivo-like models by adding oligomycin (complex III inhibitor) as positive control. Cells were treated with 25 μM NMac1, NMac24, phenformin and oligomycin, 1 mM metformin, and 100 μM Etomoxir and then, the 3D-spheroid cell sizes were assessed. NMac24 shows the most potent anticancer effect in the 3D-spheroid model of MDA-MB-231 cells, followed by phenformin, then NMac1 and oligomycin (Fig. 8d). Although phenformin and oligomycin showed higher efficacy than other classical drugs, these were withdrawn from markets because they trigger the severe lactic acidosis^11,12^. Our results suggest that NMac1 and NMac24 can reduce the tumor cell proliferation at 500-fold lower concentration to generate the same extent with metformin for mitochondrial dysfunction. These findings collectively show that NMac1 and NMac24 can cause glucose starvation-specific cell death in vitro and thus, they may target selectively cancer tissues in vivo because tumor tissues are under glucose deprived state, up to tenfold lower than normal tissues.

## Discussion

In the present study, we identified NMac1, a small molecule NDPK activator as an anti-metastatic agent, having anti-proliferative activity only under glucose deprived condition via inhibiting OXPHOS of breast cancer cell lines. We also examined the structure–activity relationships of various synthetic derivatives of NMac1 and elucidated NMac24 shows the most potent anti-proliferative activity. We investigated the mode of action of NMac1 on cell proliferation in the absence of glucose employing DNA microarray, mitochondrial activity and metabolomics studies. We found that these compounds induce mitochondrial dysfunctions, and significantly inhibit the proliferation of breast cancer cells, MDA-MB-231 in 3D-spheroid culture only under glucose starvation. These studies suggest the potential of NMac1 and NMac24 as anti-cancer agents for the metastatic breast cancer.

This study investigated the mode of action of NMac1 under glucose starvation, which is mimicking the tumor microenvironment. NMac1 and NMac24 induce the AMPK activation signaling pathway via ATP depletion by inhibiting mitochondrial complex 1 and lead the disruption of mitochondrial membrane potential (MMP) by increase of mitochondrial ROS in the absence of glucose, not in the presence of glucose (Fig. 8e). To ensure the compounds induced mitochondrial dysfunction, we compared the metabolomics and microarray analyses in response to NMac1 in the absence and presence of glucose. The significant changes in metabolomics (Fig. 5a) and DNA microarray analysis (Fig. 2) were observed by NMac1 in the absence of glucose, neither by NMac1 in the presence of glucose nor by glucose starvation itself. Metabolomics results show that NMac1 in the absence of glucose inhibits TCA cycle, protein synthesis and gluconeogenesis (Supplementary Fig. 4c). The TCA cycle disruption by NMac1 results in the decrease of intermediates in TCA cycle, including succinate, 2-oxoglutarate, malate, citrate and fumarate. In addition, accumulation of most amino acids by NMac1 is caused by the prevention of protein synthesis. NMac1 impedes the gluconeogenesis, increasing the intermediate amino acids and decreasing the UDP-glucose/galactose/glucuronate, which abrogate the compensation process in glucose deprived condition and induce cell death. Further studies are required for understanding the molecular mechanism of NMac1 acting on these processes.

To ascertain the possibility of NMac1 and NMac24 as anti-cancer agents, three-dimensional (3D) cell culture was employed for in vivo relevance. The 3D-spheroid model allows cells to grow and interact with their surroundings in all three dimensions and thus it is considered to have high in vivo relevance^50^. NMac1 and NMac24 effectively inhibit 3D spheroid tumor growth in triple-negative breast cancer cells. These results suggest the potential of NMacs for anti-cancer agents as OXPHOS inhibitors as well as anti-metastatic agents for breast cancer because these have much higher efficacy than biguanides and show no toxicity in the normal cell condition of glucose presence.

Although the most well-known metabolic reprogramming in cancer is upregulated aerobic glycolysis, mitochondrial electron transport chain (ETC) has been identified as an essential component in bioenergetics, biosynthesis, and redox control during proliferation and metastasis of cancer cells^51^. Since aggressive metastatic breast cancer cells showed the elevated OXPHOS for spreading to bone or lung, several drugs being already used clinically for non-oncologic indications such as metformin, atovaquone and arsenic trioxide are highlighted because of recent data demonstrating their potential use as OXPHOS inhibitors^2^. MDA-MB-231 cells are resistant to glucose depletion and the results obtained from metabolomics analysis showed no discernible differences in metabolite profiles between the presence and absence of glucose without NMac1 treatment. The results suggest that obvious NMac1-induced inhibition of TCA cycle, protein synthesis and gluconeogenesis under glucose starvation consequently causes cancer cell death.

In summary, this study identifies NMac1 and NMac24 as novel compounds for cancer-specific anti-proliferative activity because this activity exerts exclusively under glucose-depleted condition and describes the mode of action of NMacs, inducing mitochondrial dysfunction. This study suggests the usage of NMacs as not only anti-metastatic agents but also anti-tumor agents in breast cancer. Further studies are needed to understand the mode of action and differential sensitivities against OXPHOS inhibition of NMac1 and NMac24 between the presence and absence of glucose.

## Methods

### Materials and instrument

Monoclonal anti-α-tubulin antibody was purchased from Santa Cruz Biotechnology, monoclonal anti-AMPK, ERK, mTOR, ACC, p-AMPK, p-ERK, p-mTOR and p-ACC antibodies from Cell Signaling Technology (CST) and monoclonal anti-puromycin antibody from EMD Millipore Co. (Merck Millipore). Penicillin, streptomycin, fetal bovine serum (FBS) and trypsin were purchased from GIBCO Life Technologies Inc., EMEM (LM007-75 and LM007-87 for glucose starvation and normal glucose condition, respectively) and DMEM (LM001-56) media from Welgene Biotech Co. Each media contains same concentrations of ingredients except glucose. Media for glucose-starved condition, low-glucose condition and normal glucose condition contain 0, 500 and 1000 mg/L (0, 2.8 and 5.5 mM) of glucose, respectively.

### Cell lines

MDA-MB-231 cells (ATCC) and MCF7 cells (KCLB) were cultured in EMEM media, and MEF cells (ATCC) in DMEM media supplemented with 10% FBS, 1 mM sodium pyruvate, 100 μg/mL streptomycin and 100 units/mL penicillin G, indicated as complete media (CM) at 37 °C with 5% CO_2_.

### Intracellular ATP determination assay

Cellular ATP concentrations were assessed by ATP determination kit (A22066, Molecular Probes) according to the manufacturer’s protocol. MDA-MB-231 cells (200,000) were treated with indicated concentrations of compounds for indicated times and washed with PBS, lysed with 200 μL of hypotonic solution containing protease inhibitor cocktail, centrifuged at 4000 *g* for 15 min at 4 °C. Cell lysates (5 μL) were incubated with 100 μL standard reaction solution (0.5 mM d-luciferin, 6.25 μg/mL firefly luciferase and 0.5 mM DTT in reaction buffer). Luminescence was measured at 560 nm by SpectraMax190 (E-innotech).

### xCELLigence real-time cell proliferation assay

xCELLigence RTCA SP (ACEA Bioscience) was employed to assay the cell proliferation real-time, which analyzes magnitude of impedance dependently on attached surfaces of the cells. MDA-MB-231, MCF7 and MEF cells (7500) were suspended in 75 μL of media, seeded and grown on RTCA plate. After 24 h, media was changed to indicated chemical-containing EMEM-CM or DMEM-CM media with or without glucose and cell proliferation was monitored at 37 °C with 5% CO_2_.

### Microarray analysis

Global gene expression in MDA-MB-231 cells exposed to control DMSO (Ctrl) and NMac1 in the presence (Glc(+)) and absence (Glc(−)) of glucose were evaluated using microarray analysis. Cells were exposed to 10 μM NMac1 in Glc(+) and 5 μM NMac1 in Glc(−) EMEM-CM for 16 h. Total RNAs of cells harvested at each sample were isolated using Trizol reagent (Invitrogen). RNA purity and integrity were evaluated by ND-1000 Spectrophotometer (NanoDrop), Agilent 2100 Bioanalyzer (Agilent Technologies). Total RNA was amplified and purified using TargetAmp-Nano Labeling Kit for Illumina Expression BeadChip (EPICENTRE) to yield biotinylated cRNA according to the manufacturer’s instructions. Briefly, 300 ng of total RNA was reverse transcribed to cDNA using a T7 oligo(dT) primer. Second-strand cDNA was synthesized, in vitro transcribed, and labeled with biotin-NTP. After purification, the cRNA was quantified using the ND-1000 Spectrophotometer (NanoDrop).

Labeled cRNA samples (750 ng) were hybridized to each Human HT-12 v4.0 Expression Beadchip for 18 h at 58 °C, according to the manufacturer's instructions (Illumina, Inc.). Detection of array signal was carried out using Amersham fluorolink streptavidin-Cy3 (GE Healthcare Bio-Sciences) following the bead array manual. Arrays were scanned with an Illumina bead array Reader confocal scanner according to the manufacturer's instructions. The quality of hybridization and overall chip performance were monitored by visual inspection of both internal quality control checks and the raw scanned data. Raw data were extracted using the software provided by the manufacturer [Illumina GenomeStudio v2011.1 (Gene Expression Module v1.9.0)]. Array probes transformed by logarithm and normalized by quantile method. Data analysis was performed by Macrogen INC.

Statistical significance of the expression data was determined using LPE test and fold change in which the null hypothesis was that no difference exists among groups. False discovery rate (FDR) was controlled by adjusting *p*-value using Benjamini–Hochberg algorithm. For a differentially expressed gene (DEG) set, hierarchical cluster analysis was performed using complete linkage and Euclidean distance as a measure of similarity. All data analysis and visualization of differentially expressed genes was conducted using R3.0.2 (http://www.r-project.org). Sample similarities were visualized and grouped using hierarchical clustering (Euclidean method, complete linkage). Protein network was analyzed using STRING (string-db.org).

### Western blot analysis

Proteins were separated by SDS-PAGE and transferred to PVDF membrane. Each protein was detected using primary and secondary antibodies. Immunoblots were analyzed with Amersham imager 600 (GE healthcare) using ECL Prime Western Blotting Detection Reagent (GE Healthcare) as chemiluminescent substrates.

### Mitochondrial membrane potential (MMP) measurement

All experiments with flow cytometer were performed with 10,000 cells detection. MDA-MB-231 cells (200,000) were treated with indicated concentrations of chemicals and stained with 100 nM of TMRM (Molecular Probes, Invitrogen) in Hanks balanced salt solution (HBSS) for 30 min at 37 °C. After staining, cells were trypsinized and re-suspended in cold HBSS. Then, cells were separated by 40 μm pore size cell strainer (SPL Life Sciences). TMRM fluorescence was analyzed with FL2 channel of FACS Calibur (BD Biosciences). Non-stained cells were used to detect background staining. Geometric mean fluorescence intensity (MFI) of TMRM was used to calculate relative MFI ratio.

### Oxygen consumption rate (OCR) and extracellular acidification rate (ECAR) measurement

Oxygen consumption rate (OCR) and extracellular acidification rate (ECAR) were assessed with XF96 Seahorse Bioscience Extracellular Flux Analyzer (Agilent Technologies) according to manufacturer’s protocol. In brief, MDA-MB-231 cells seeded a day before assay were sequentially injected with indicated concentrations of compounds, 1 μM oligomycin, 0.5 μM FCCP and 0.5 μM rotenone/antimycin A with 30 min intervals. The values were detected every 10 min using the Flux Analyzer.

### Confocal microscopy

MDA-MB-231 cells grown on SecureSlip coverslips (Sigma-Aldrich) were fixed with 4% paraformaldehyde (PFA), then permeabilized with 0.1% Triton X-100 in HBSS for 15 min and washed with HBSS. Cells were incubated with 3% BSA in HBSS for 1 h and then with TOM20 primary antibody diluted for 1:100 overnight at 4 °C. Cells washed three times with HBSS were incubated with fluorochrome-conjugated secondary antibody diluted for 1 or 2 h at RT in the dark. Coverslips were mounted on a glass slide after DAPI in Fluoroshield Mounting Medium was added to the top of the cells. Fluorescence images were taken with Zeiss LSM510 META with IMARIS (Zeiss).

### Crystal violet staining

MDA-MB-231 cells cultured on 24-well plate for 48 h were treated with 10 μM of chemicals for 24 h and fixed with 4% PFA for 10 min. Then, cells were washed with PBS, stained with 0.25% crystal violet in 12.5% methanol for 10 min. Finally, cells were washed once with PBS and 3 times with distilled water.

### Calculation of IC_50_

MDA-MB-231 cells were cultured on RTCA plate and monitored with xCELLigence RTCA for 24 h. Cells were treated with 0.6, 1.25, 2.5, 5, 10 and 20 μM of chemicals in EMEM-CM media under glucose starvation. IC_50_ values were calculated by relative cell proliferation index after 24 h of chemical treatment. The data were analyzed using Prism 9 (GraphPad software).

### NDPK enzyme assay

Mixture of recombinant Nm23-H1 (5 ng) and 10 µM GTP (or UTP) in NDPK assay buffer (20 mM HEPES, 3 mM MgCl_2_) was pre-incubated with NMac1 or other analogue chemicals at RT for 10 min. Then, enzyme reaction was performed by adding final reaction concentration of 5 μM ADP and incubating for 1 min. Enzyme reaction was stopped by inactivating the samples at 95 °C for 10 min and measured the produced ATP as luminescence with ATP determination kit (A22066, Molecular Probes) using SpectraMax190 (E-innotech).

### Mitochondrial reactive oxygen species (ROS) measurement

MDA-MB-231 cells (200,000) cultured on 35 mm cell culture plate (Thermo Fisher Scientific) for 24 h, and treated with chemicals for indicated time, were stained with 2 μM mitoSOX (Invitrogen, Carlsbad) for 20 min at 37 °C. Cells were then trypsinized, harvested and re-suspended with 300–500 μL HBSS and separated with 40 μm pore size cell strainer (SPL Life Sciences). MitoSOX fluorescence was analyzed by flow cytometer with FL2 channel using BD FACS Calibur (BD Biosciences). Non-stained cells were used to detect background staining.

### Complex I enzyme activity assay

The mitochondrial complex 1 activity of MDA-MB-231 cells were measured by Complex I Enzyme Activity Assay Kit (Ab109721, Abcam) according to the manufacturer’s protocol. Briefly, MDA-MB-231 cell lysates were collected by homogenizing with 31G syringe. Based on BCA assay, proteins in cell lysates were diluted to 5–6 mg/mL in PBS. After additional mitochondrial membrane fragmentation by detergent supplied in kit for 10–30 min, cell debris were eliminated by centrifugation at 14,000 *g*, 4 °C for 20 min. The supernatant, which contains complex I enzyme, was incubated with DMSO as control or indicated concentrations of NMac1 for 3 h at RT and loaded on a 96-well plate. The absorbance at 450 nm was detected real-time for 1.5 h.

### Metabolic analysis using NMR

MDA-MB-231 and MCF7 cells (5 samples in each group) were treated 10 μM NMac1 with or without glucose for 8 h. Metabolites were extracted from cells with 80% methanol (v/v). ^1^H-NMR spectra were measured using an 800-MHz NMR instrument (Bruker BioSpin). A NOESYPRESAT pulse sequence was applied to suppress the residual water signal. For each sample, 512 transients were collected into 64,000 data points using a spectral width of 16,393.4 Hz with a relaxation delay of 4.0 s and an acquisition time of 2.00 s. All acquired ^1^H-NMR spectra were phased and baseline-corrected using TopSpin 3.1 software (Bruker BioSpin) and AMIX (Bruker BioSpin). Resonance assignments for cell metabolites were accomplished using the 800 MHz library of Chenomx NMR Suite Version 7.1 (Chenomx).

### Three-dimensional (3D) tumor invasion assay

Spheroid cell culture with MDA-MB-231 cells was performed in 2.5% matrigel (Corning Life Sciences). Briefly, 7500 cells in 75 μL culture media were suspended to 75 μL of 5% matrigel, then, centrifuged at 300*g* for 10 min. Cells were incubated 3 days for spheroid initiation in cell carrier spheroid ULA 96-well microplate (Perkin Elmer). After the initiation of spheroids, indicated concentrations of chemicals were treated. Spheroid invasion was assessed by calculating radius of spheroid employing microscopic images.

### Puromycin-based protein synthesis assay

Puromycin incorporation during protein synthesis^52^ was used to measure newly synthesized proteins. MDA-MB-231 cells were treated with 0.025% DMSO as control, 10 or 20 μM of NMac1 for 8 h. During NMac1 treatment, 0.5 μg/mL puromycin was added after 6 h of NMac1 treatment and incubated for additional 2 h. 10 μg/mL cycloheximide (CHX), a protein synthesis inhibitor, was treated for 8 h as background control of puromycin incorporation. Then, incorporated puromycin level in cell lysates which represents newly synthesized proteins, were detected by western blot analysis using anti-puromycin antibody (MABE343, EMD Millipore Corp.).

### Statistical analysis

Data were analyzed with Student’s *t* test for comparisons between two groups to determine the statistical significance (*p* value). *p* < 0.05 was considered as statistically significant.

## Supplementary Information

Supplementary Information.
