# Supplementary material for: Nm23-H1 activator phenylbutenoid dimer exerts cytotoxic effects on metastatic breast cancer cells by inducing mitochondrial dysfunction only under glucose starvation
Source: Sci Rep. 2021 Dec 7;11:23549. doi: 10.1038/s41598-021-02729-7 (PMC8651694; doi:10.1038/s41598-021-02729-7)
Supplement: Supplementary file 1 — Supplementary Information. [file 41598_2021_2729_MOESM1_ESM.pdf]

Supplementary Information

Nm23-H1 activator Phenylbutenoid dimer Exerts Cytotoxic Effects on Metastatic Breast Cancer Cells by Inducing Mitochondrial Dysfunction only under Glucose Starvation

Bokyung Kim[1], Jae-Jin Lee[1], Ji Soo Shin[2], Ji-Wan Suh[1], Sunhee Jung[3], Geum-Sook Hwang[3], Hee-Yoon Lee[2*], Kong-Joo Lee[1*]

Contents

Supplementary Table 1……………………………………………………..……S-1
Supplementary Table 2……………………………………….……………..S-2
Supplementary Figure 1…………………………………………….……...S-3
Supplementary Figure 2…………………………………………………....S-4
Supplementary Figure 3…………………………………….……………....S-5
Supplementary Figure 4……………………………………………………S-6
Supplementary Figure 5……………………………………………………S-7
Supplementary Figure 6………………………………………..………...S-8
Supplementary Figure 7…………………………………………..……...S-9
Supplementary Figure 8……………………………………………..….S-12

Supplementary Table 1 Differentially expressed gene (DEG)s in Group 2 of DNA microarray. Upregulated and downregulated genes are colored in red and green, respectively, based on │FC│≥2.0.

Gene symbol	Fold change	Adjusted P value
HSPA6	13.0	0.0
HSPA7	6.2	0.0
GDF15	5.8	0.0
HSPA1A	5.3	0.0
LOC85390	5.2	0.0
SIK1	5.2	0.0
LOC85389	4.3	0.0
FBXO32	3.6	0.0
DNAJB1	3.3	0.0
CTH	3.2	0.0
RP5-1022P6.2	3.2	0.0
RHOB	3.1	0.0
SNORA8	2.9	0.0
ARRDC3	2.8	0.0
ABL2	2.7	0.0
CCNG2	2.7	0.0
CEBPD	2.7	0.0
CLK1	2.7	0.0
FBXO32	2.6	0.0
JMY	2.6	0.0
ID2	2.6	0.0
PIM1	2.6	0.0
RNU11	2.6	0.0
DUSP10	2.6	0.0
SGK1	2.5	0.0
SNORD21	2.5	0.0
DDIT4	2.5	0.0
NGDN	2.5	0.0
RNU1-5	2.4	0.0
IL6	2.4	0.0
SNAI2	2.4	0.0
FAM160B1	2.4	0.0
CDCP1	2.4	0.0
SLC2A3	2.4	0.0
SGK1	2.4	0.0
HMOX1	2.4	0.0
RN5S9	2.3	0.0
RNU1-3	2.3	0.0
DUSP1	2.3	0.0
BRWD1	2.3	0.0
DNAJB9	2.3	0.0
RNU1G2	2.3	0.0
SDHALP1	2.3	0.0
AVPI1	2.3	0.0
PHF17	2.3	0.0
CLK1	2.3	0.0
FBXL20	2.3	0.0
PPAP2B	2.2	0.0
BHLHB2	2.2	0.0
TRIB3	2.2	0.0
SNORD35B	2.2	0.0
SGK	2.2	0.0
MYLIP	2.2	0.0
CHMP1B	2.2	0.0
SLC6A6	2.2	0.0
08-Mar	2.2	0.0
WWP2	2.2	0.0
EEA1	2.2	0.0
ARL4A	2.2	0.0
FAM21B	2.1	0.0
FLJ10996	2.1	0.0
ID2	2.1	0.0
FAM46A	2.1	0.0
HSPBL2	2.1	0.0
FOSL2	2.1	0.0
HSN2	2.1	0.0
VASN	2.1	0.0
NNMT	2.1	0.0
WDR33	2.1	0.0
HSPA1B	2.1	0.0
PPM1A	2.1	0.0
SNORA25	2.1	0.0
PHF17	2.1	0.0
SNORA33	2.0	0.0
HIF1A	2.0	0.0
SLC30A1	2.0	0.0
CCNG2	2.0	0.0
WDR33	2.0	0.0
C5orf41	2.0	0.0
PPAP2B	2.0	0.0
NET1	2.0	0.0
USP48	2.0	0.0
RGS2	2.0	0.0
PAWR	2.0	0.0
SNORD69	2.0	0.0
CSTF3	-2.0	0.0
LOC642921	-2.0	0.0
NEIL3	-2.0	0.0
RGS4	-2.0	0.0
SGOL2	-2.0	0.0
TMEM138	-2.0	0.0
NUPL2	-2.0	0.0
BAPX1	-2.0	0.0
ZNF175	-2.0	0.0
SLC4A7	-2.0	0.0
ROR1	-2.0	0.0
FUT4	-2.0	0.0
ZNRF3	-2.0	0.0
AMD1	-2.1	0.0
LOC113386	-2.1	0.0
STARD13	-2.1	0.0
LOC100131261	-2.1	0.0
BIRC3	-2.1	0.0
FAM111A	-2.1	0.0
ZNF114	-2.1	0.0
C2orf44	-2.1	0.0
ZNF702P	-2.1	0.0
RASSF1	-2.1	0.0
FLJ44342	-2.2	0.0
BCDIN3D	-2.2	0.0
CDCA8	-2.2	0.0
E2F8	-2.2	0.0
MKKS	-2.2	0.0
ARGLU1	-2.2	0.0
FAM111A	-2.2	0.0
LOC401431	-2.2	0.0
LOC148413	-2.2	0.0
LCMT2	-2.2	0.0
HYLS1	-2.2	0.0
PRPF4B	-2.2	0.0
FAR1	-2.3	0.0
NCOA5	-2.3	0.0
DEM1	-2.3	0.0
CCNE2	-2.3	0.0
ZNF594	-2.3	0.0
RBBP4	-2.3	0.0
RUNX1	-2.3	0.0
CDC7	-2.3	0.0
E2F2	-2.3	0.0
AP3M2	-2.4	0.0
MYEOV	-2.4	0.0
FBXO5	-2.4	0.0
PNRC2	-2.4	0.0
ZNF789	-2.5	0.0
PCF11	-2.5	0.0
ZNF302	-2.5	0.0
RARA	-2.5	0.0
EIF5	-2.6	0.0
CCNF	-2.6	0.0
LDLR	-2.7	0.0
MAT2A	-2.7	0.0
SOCS4	-2.7	0.0
ZC3HAV1	-2.7	0.0
LOC100216001	-2.8	0.0
C15orf52	-2.9	0.0
RPPH1	-3.0	0.0
C1orf110	-3.1	0.0
MIR1978	-3.4	0.0

Supplementary Table 2 Activities of Nm23 activators in anti-metastasis and anti-proliferation.

	Structure	NDPK Activity	Anti-proliferative Activity		Structure	NDPK Activity	Anti-proliferative Activity
NMac1	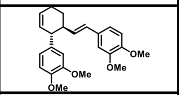	4	O (IC_{50} 7.20 μM)	NMac20	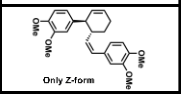	1.9	X
NMac2	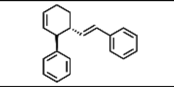	inhibitor	X	NMac21	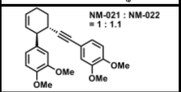	Inhibitor	O (IC_{50} 6.28 μM)
NMac3	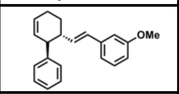	inhibitor	X	NMac22	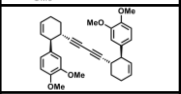	2.5	X
NMac4	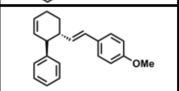	inhibitor	X	NMac23	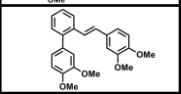	4.7	X
NMac5	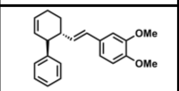	inhibitor	X	NMac24	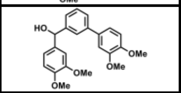	2.8	O (IC_{50} 2.46 μM)
NMac6	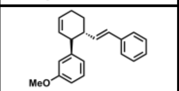	Inhibitor	X	NMac25	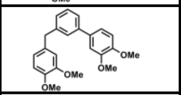	1.4	O (IC_{50} 5.04 μM)
NMac7	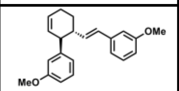	Inhibitor	X	NMac26	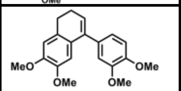	1.2	X
NMac8	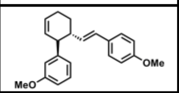	Inhibitor	X	NMac27	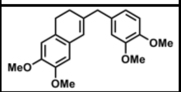	1	X
NMac9	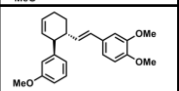	Inhibitor	X	NMac28	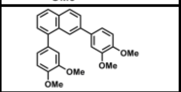	4.1	X
NMac10	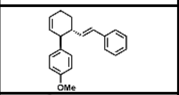	Inhibitor	X	NMac29	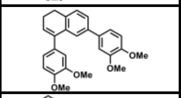	2.6	X
NMac11	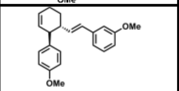	Inhibitor	X	NMac30	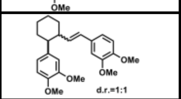	2.4	X
NMac12	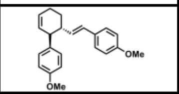	Inhibitor	X	NMac31	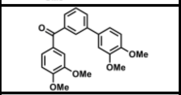	1.7	X
NMac13	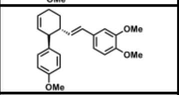	Inhibitor	X	NMac32	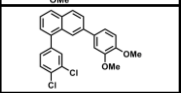	inhibitor	X
Nmac14	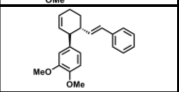	Inhibitor	X	NMac33	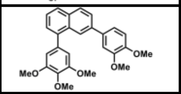	2.2	X
NMac15	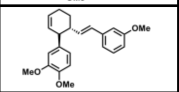	Inhibitor	X	NMac34	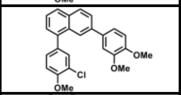	inhibitor	X
NMac16	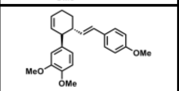	Inhibitor	X	NMac35	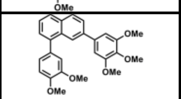	--	X
NMac17 (NMac1 isomer)	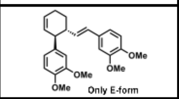	4	O	NMac36	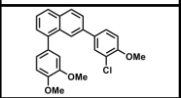	--	X
NMac18	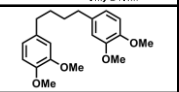	1.7	X	NMac37	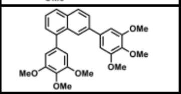	--	X
NMac19	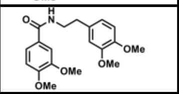	1.1	X	NMac38	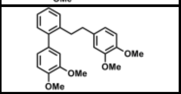	6.6	X

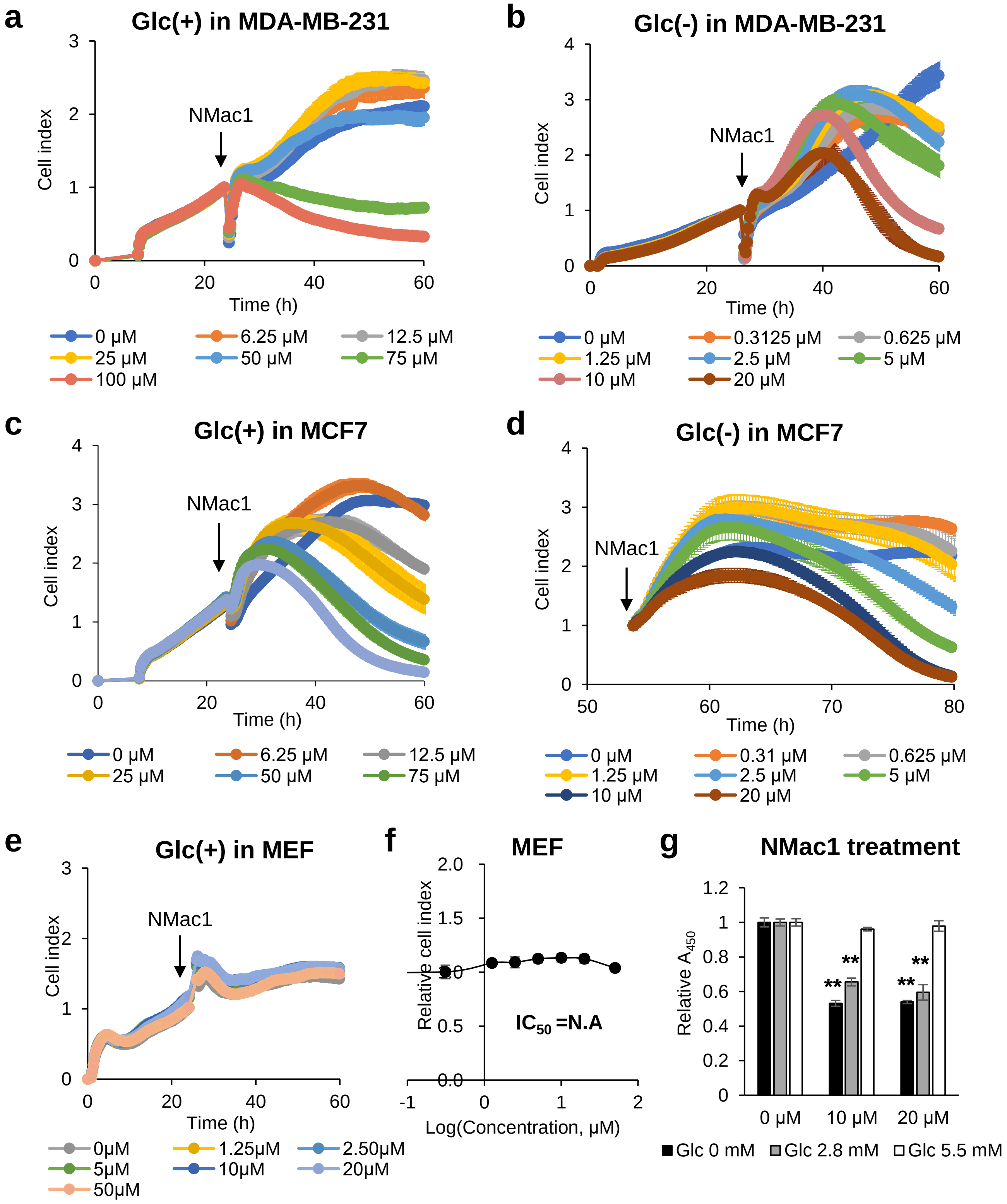

Supplementary Figure 1 Proliferative effect of NMac1 in another breast cancer cell line MCF7 and normal cell line MEF. Effect of NMac1 under glucose (a,c) presence and (b,d) absence on real-time cell proliferation in MDA-MB-231 (a,b) and MCF7 (c,d) breast cancer cell line. (e, f) Effect of NMac1 on real-time cell proliferation in MEF normal cell line under glucose presence. (g) Effect of NMac1 on cell proliferation in MDA-MB-231 cell line with glucose-gradient condition. Error bars represent SD (n=4). * $p<0.05$, ** $p<0.01$.

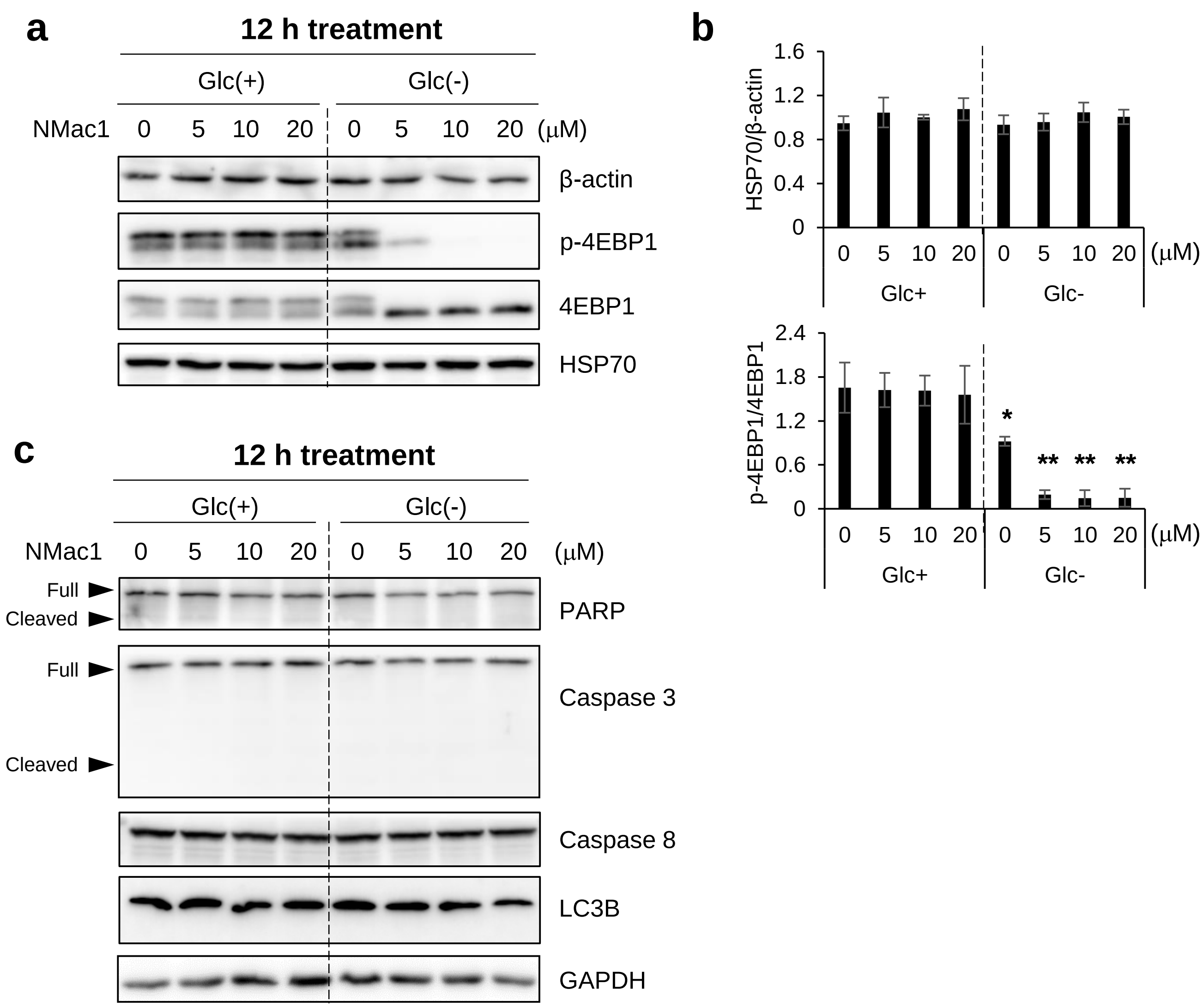

Supplementary Figure 2 Protein expression levels of the genes identified to be significantly changed in DNA microarray. (a) Signaling of stress responsive proteins that were upregulated in DNA microarray were detected in western blot analysis. (b) Each blot was quantified by Multigauge ver.3.0. MDA-MB-231 cells were treated indicated concentrations of NMac1 for 12 h under glucose presence or absence. Error bars represent SD (n=3). * $p<0.05$, ** $p<0.01$. (c) Representative immunoblots of autophagy and apoptosis markers. Full-length blots are presented in Supplementary Figure 8.

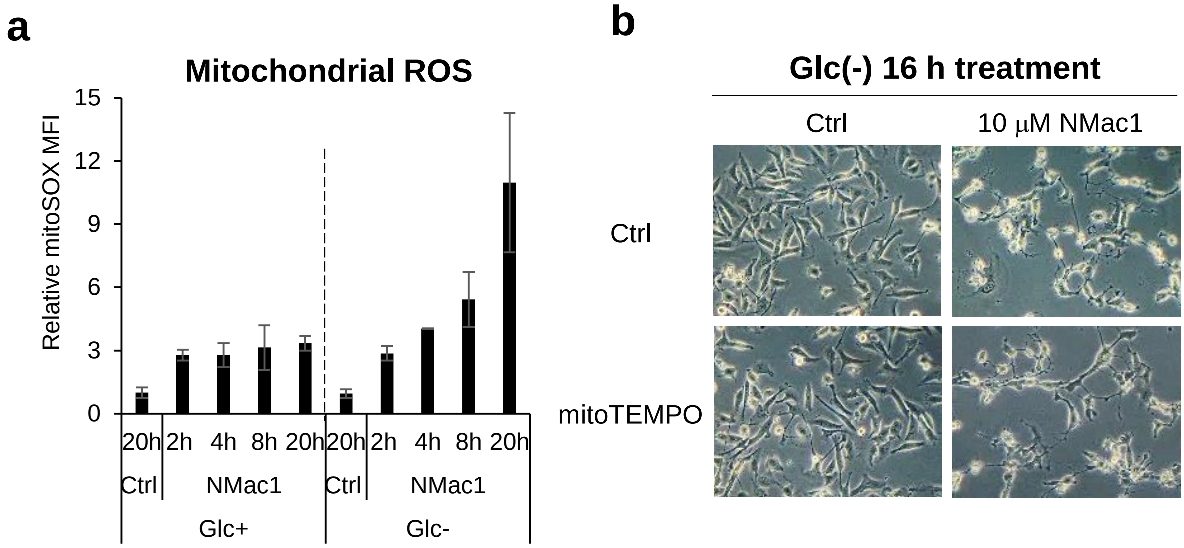

Supplementary Figure 3 Effect of scavenging mitochondrial ROS elevation. (a) Relative mitochondrial ROS level by time-dependent NMac1 treatment. MDA-MB-231 cells were treated 10 μM NMac1 and stained with 2 μM MitoSOX for 20 min. 10,000 cells were detected using FACS Calibur (BD Biosciences, USA). Error bars represent SD (n=3). (b) Representative images of cell morphology by NMac1 treatment with or without mitochondrial ROS scavenger, mitoTEMPO. MDA-MB-231 cells were treated 10 μM NMac1 with or without 50 μM mitoTEMPO for 16 h.

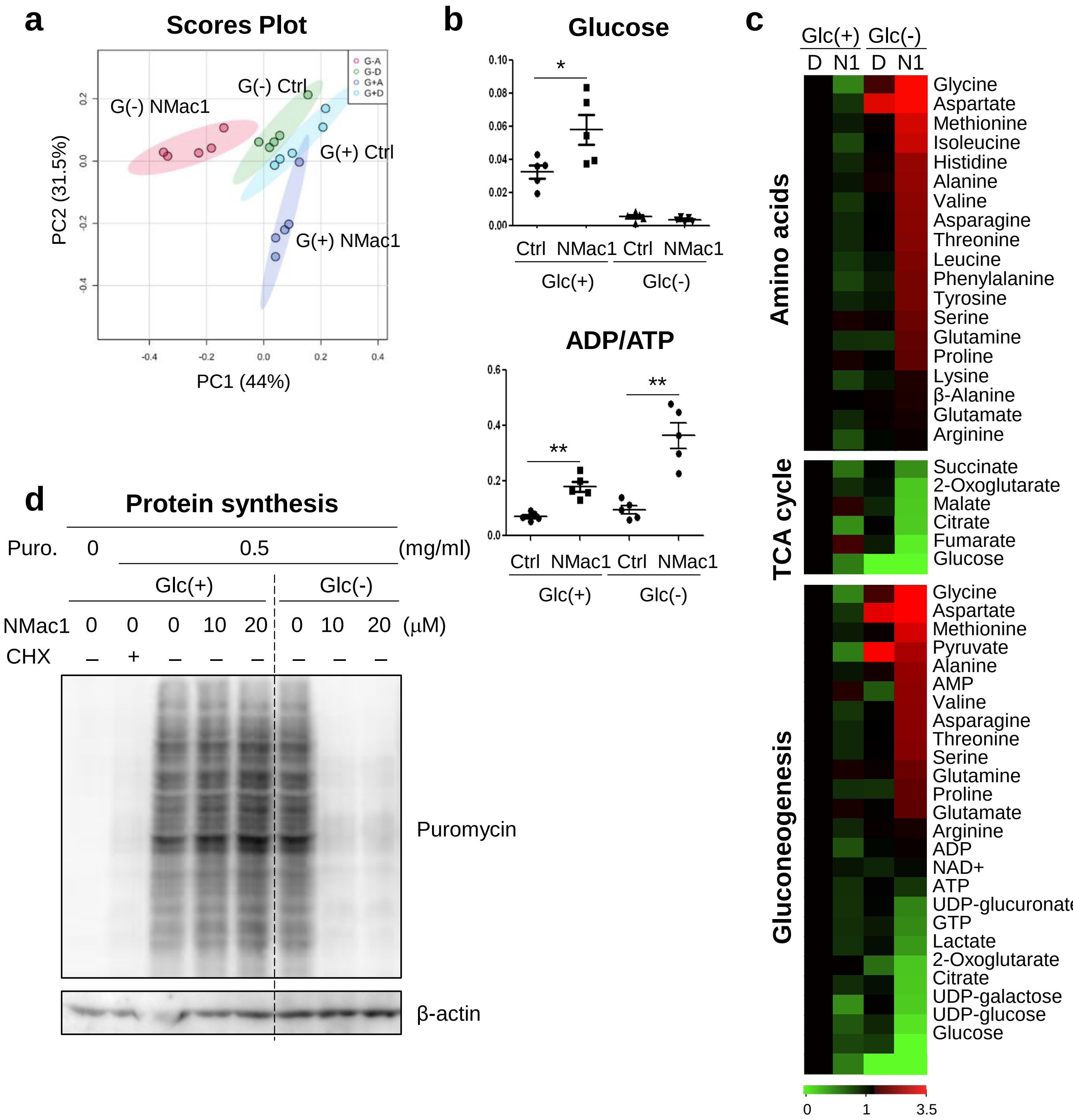

Supplementary Figure 4 Metabolomics analysis and protein synthesis in MDA-MB-231 cells. (a) Scores plot of multivariate unsupervised principal component analysis (PCA) based on metabolic analysis samples of 10 μM NMac1 treatment for 8 h. (b) Glucose and ADP/ATP levels in each group of metabolic analysis samples. * $p<0.05$, ** $p<0.01$. (c) Heatmap of metabolites related to each indicated pathway. The upregulated and downregulated metabolites are in red and green, respectively. The intensity of color represents gap of difference. (d) Representative images of western blot analysis from puromycin assay. Each indicated concentration of NMac1 was treated for 8 h and puromycin was treated for 2 h, which was added after 6 h of NMac1 treatment. 10 μg/ml cycloheximide (CHX) was used as background incorporation of puromycin. * $p<0.05$, ** $p<0.01$. Full-length blots are presented in Supplementary Figure 8.

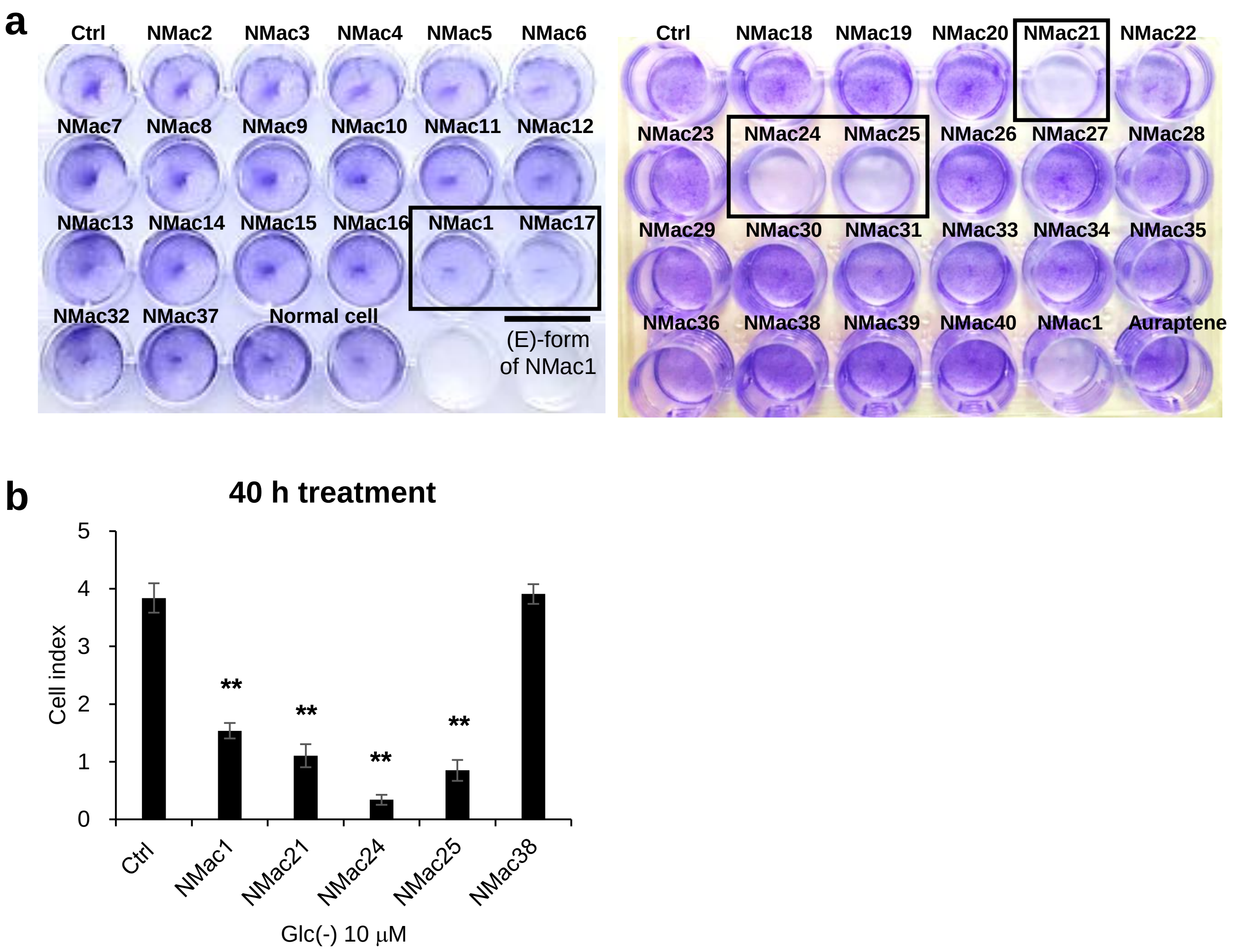

Supplementary Figure 5 Screening anti-proliferative activity of NMac1 analogues. (a) Representative images of cell viability assay by crystal violet staining. MDA-MB-231 cells were treated with 10 μM of indicated chemicals in EMEM media without glucose for 24 h. (b) Cell proliferation of MDA-MB-231 cells were measured using xCELLigence RTCA. Cells were treated with 10 μM of indicated chemicals under glucose starvation for 40 h. Error bars represent SD (n=3). * $p<0.05$, ** $p<0.01$

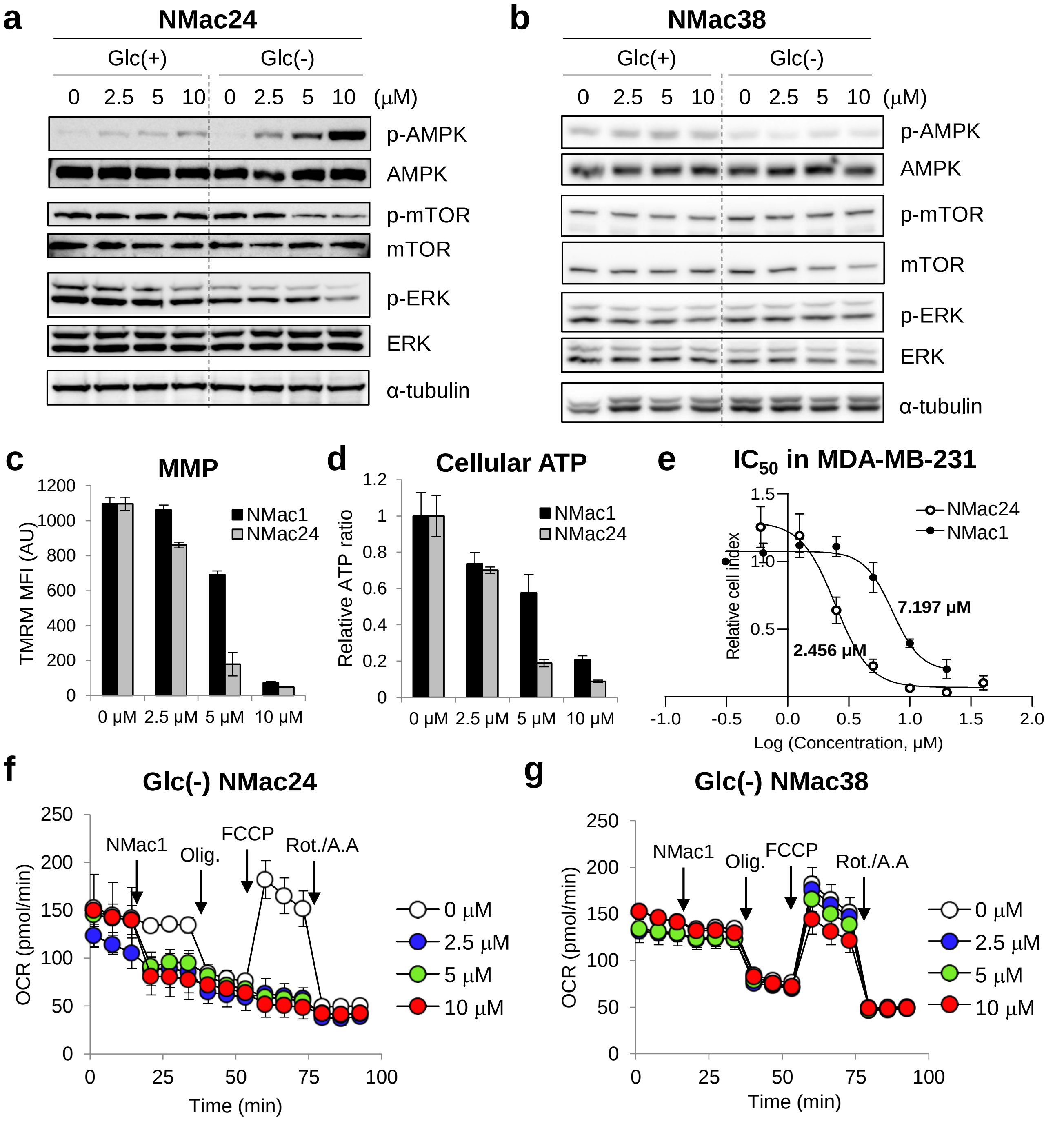

Supplementary Figure 6 Effect of NMac24 and NMac38. Representative immunoblots of AMPK/mTOR/ERK signaling with concentration-dependent (a) NMac24 or (b) NMac38 treatment. MDA-MB-231 cells were treated 0, 2.5, 5 or 10 μM of NMac24 or NMac38 under glucose presence or absence for 12 h. (c) MMP and (d) cellular ATP level of NMac1 and NMac24 treated MDA-MB-231 cells in concentration-dependent manner for 16 h. (e) IC_{50} values were calculated using Prism 9 (GraphPad, USA) based on concentration-dependent cell index measured by xCELLigence RTCA. Error bars represent SD (n=3). Full-length blots are presented in Supplementary Figure 8. (f,g) OCR of MDA-MB-231 cells were monitored with Seahorse XF96 analyzer (Agilent Technologies Inc.). NMac24 or NMac38 (0, 2.5, 5 and 10 μM), oligomycin (1 μM), FCCP (0.5 μM) and rotenone/antimycin A (0.5 μM) were injected sequentially at each time point. Error bars represent SE (n = 4).

a

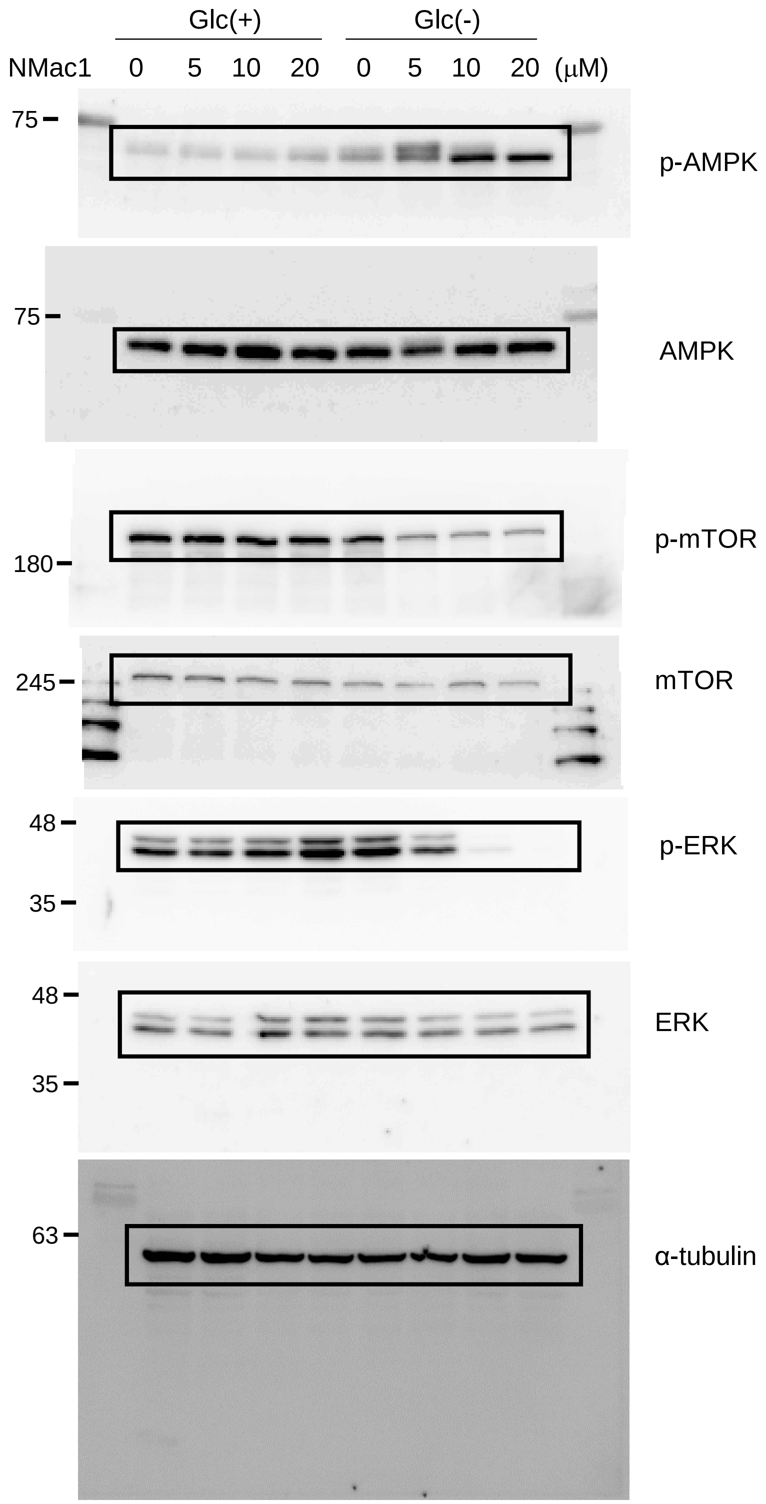

Supplementary Figure 7 Gels used in result Figures. Western blots of Figure (a) 3A, (b) 3C and (c) 7E complete blots with areas cropped for presentation. Each border line (black box) represents the same origin blot. Numbers (Left) represent the size (kDa) of each protein marker.

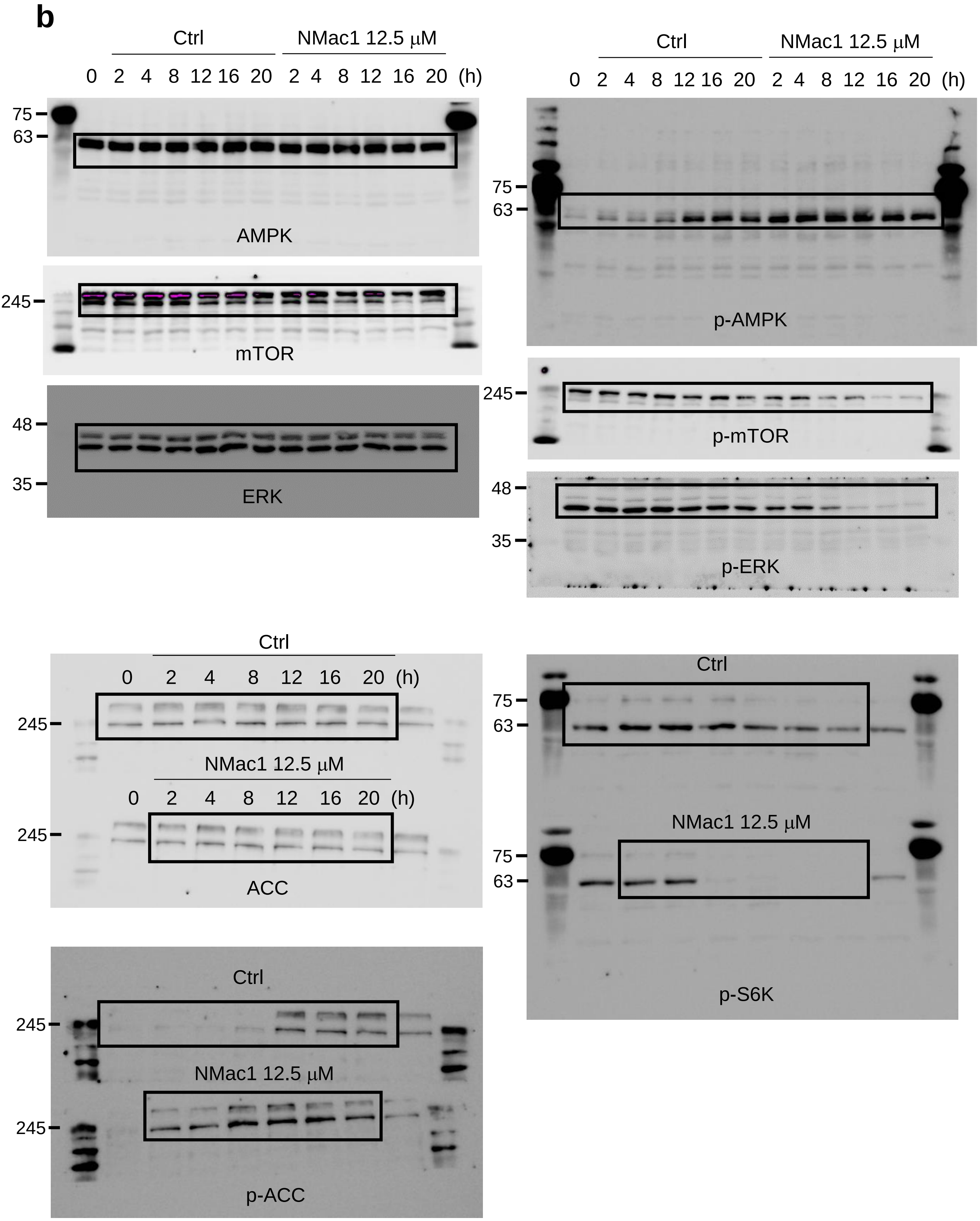
b
Ctrl
NMac1 12.5 μM
0 2 4 8 12 16 20 2 4 8 12 16 20 (h)
75
63
AMPK
245
mTOR
48
35
ERK
p-AMPK
p-mTOR
p-ERK
0 2 4 8 12 16 20 (h)
ACC
p-ACC
p-S6K

c

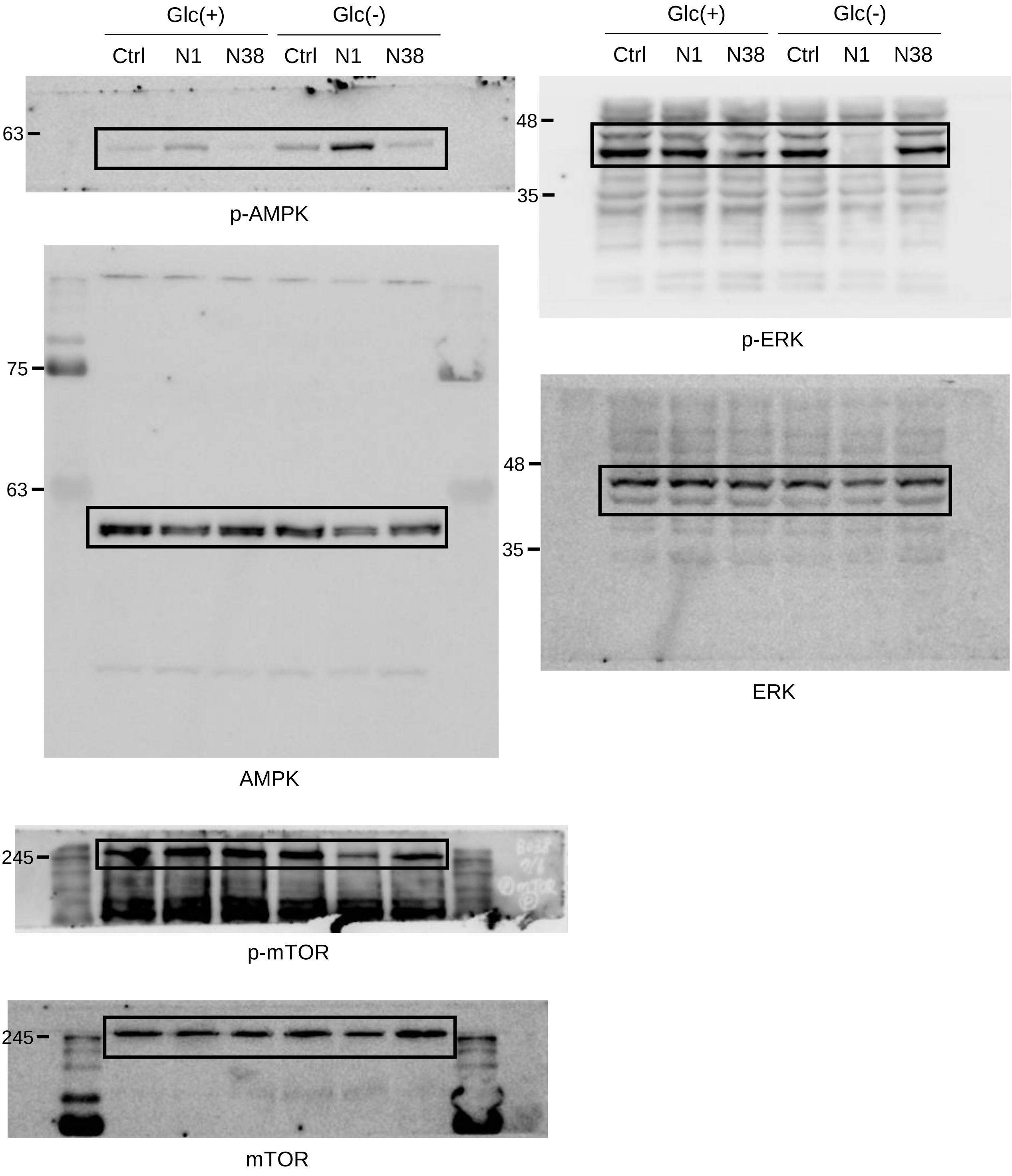
Glc(+)
Glc(-)
Ctrl
N1
N38
Ctrl
N1
N38
63
p-AMPK
75
63
AMPK
48
35
p-ERK
48
35
ERK
245
p-mTOR
245
mTOR

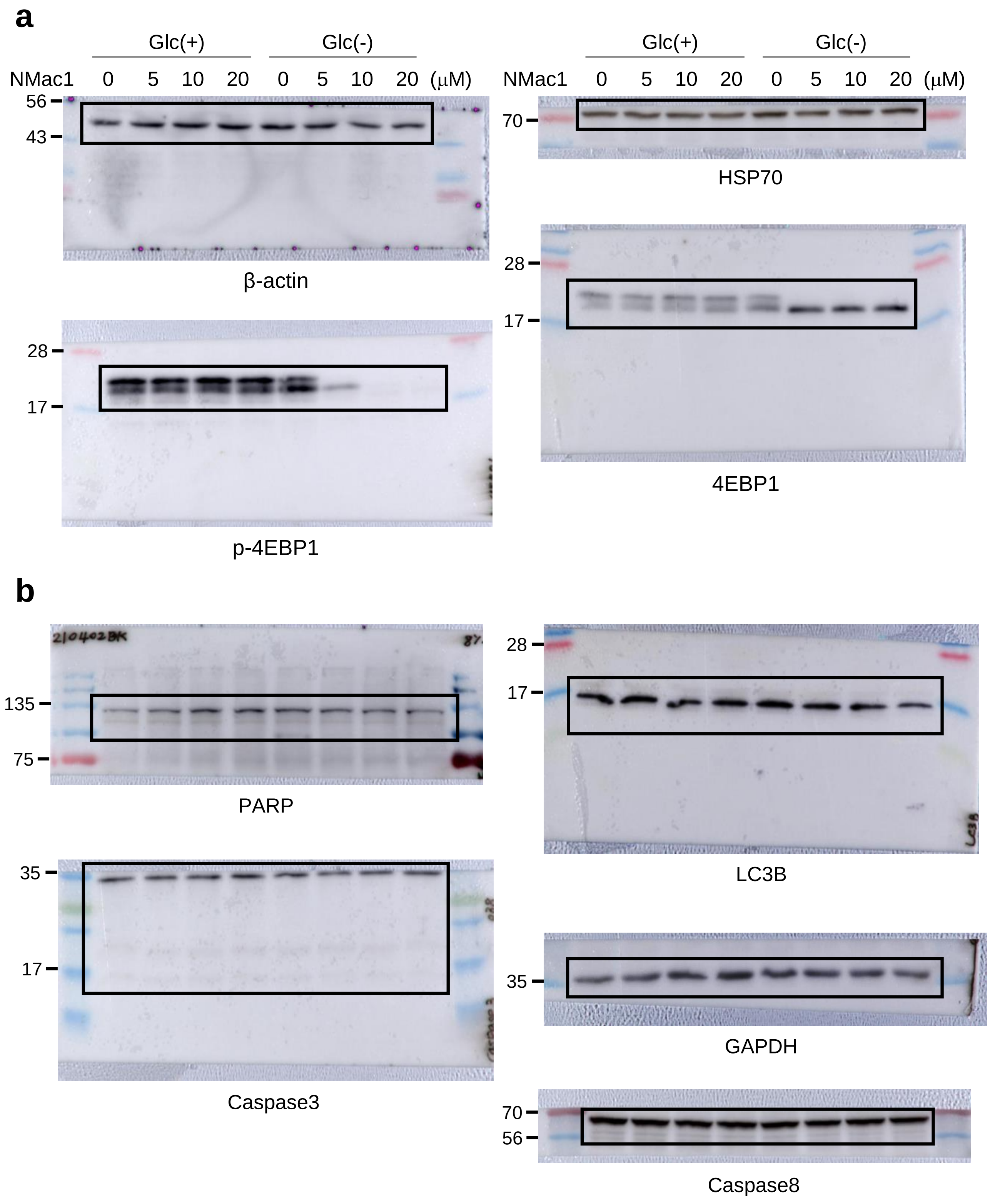

Supplementary Figure 8 Gels used in Supplementary Figures. Western blots of Supplementary Figure (a) 2A, (b) 2C, (c) 4D, (d) 6A and (e) 6B complete blots with areas cropped for presentation.

c

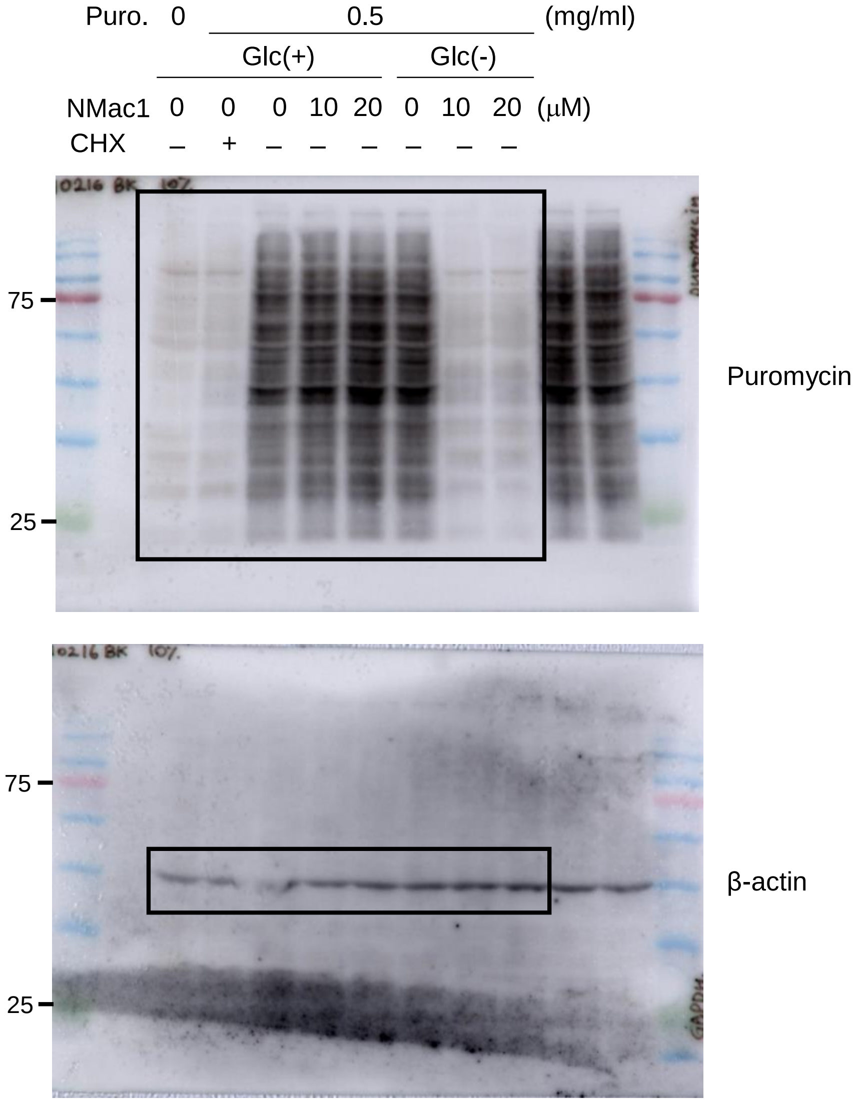
Puro. 0 0.5 (mg/ml)
Glc(+) Glc(-)
NMac1 0 0 0 10 20 0 10 20 (μM)
CHX – + – – – – – –
75
25
Puromycin
75
25
β-actin

d

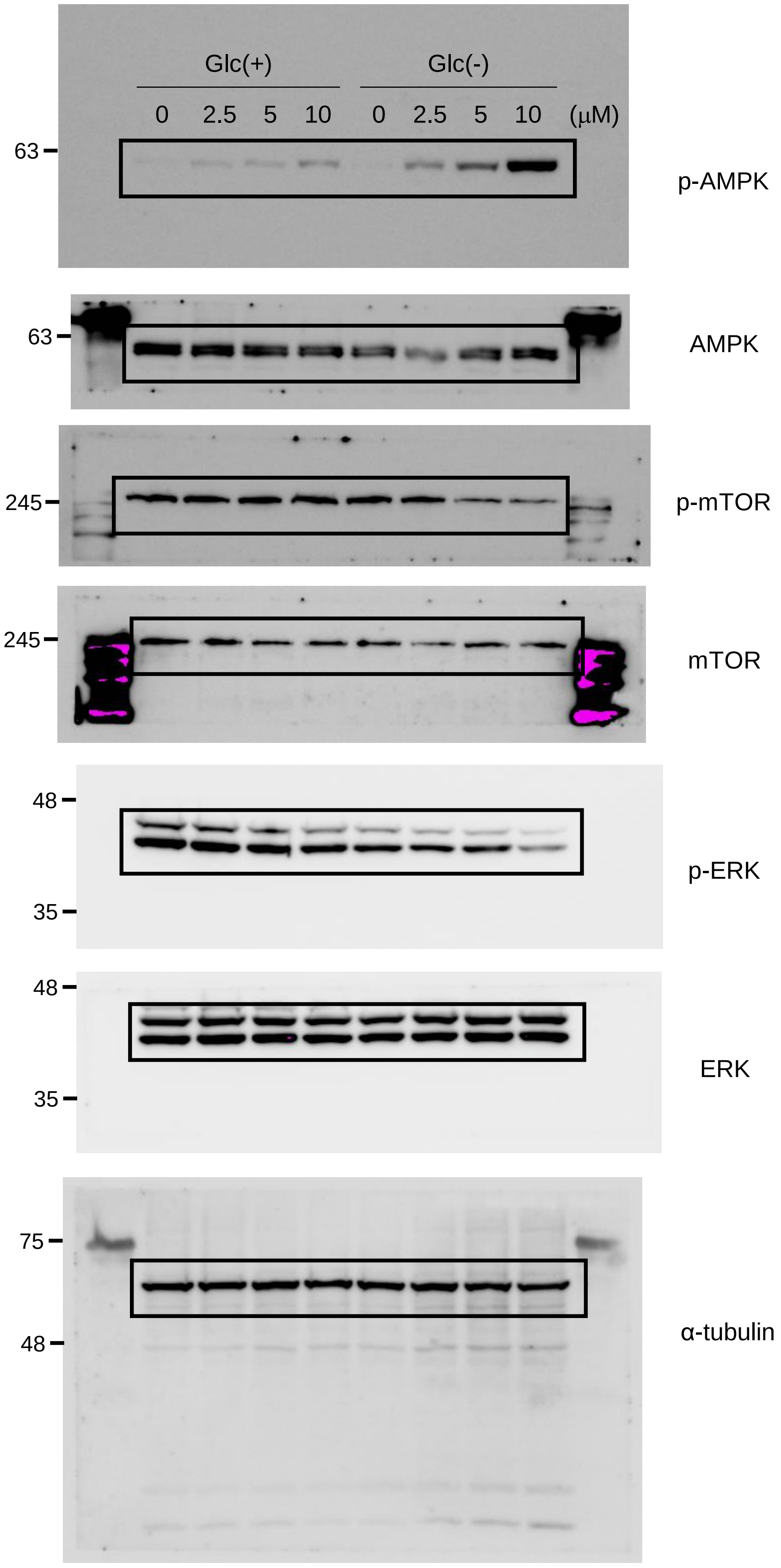
Glc(+)
Glc(-)
0 2.5 5 10 0 2.5 5 10 (μM)
63
p-AMPK
63
AMPK
245
p-mTOR
245
mTOR
48
35
p-ERK
48
35
ERK
75
48
α-tubulin

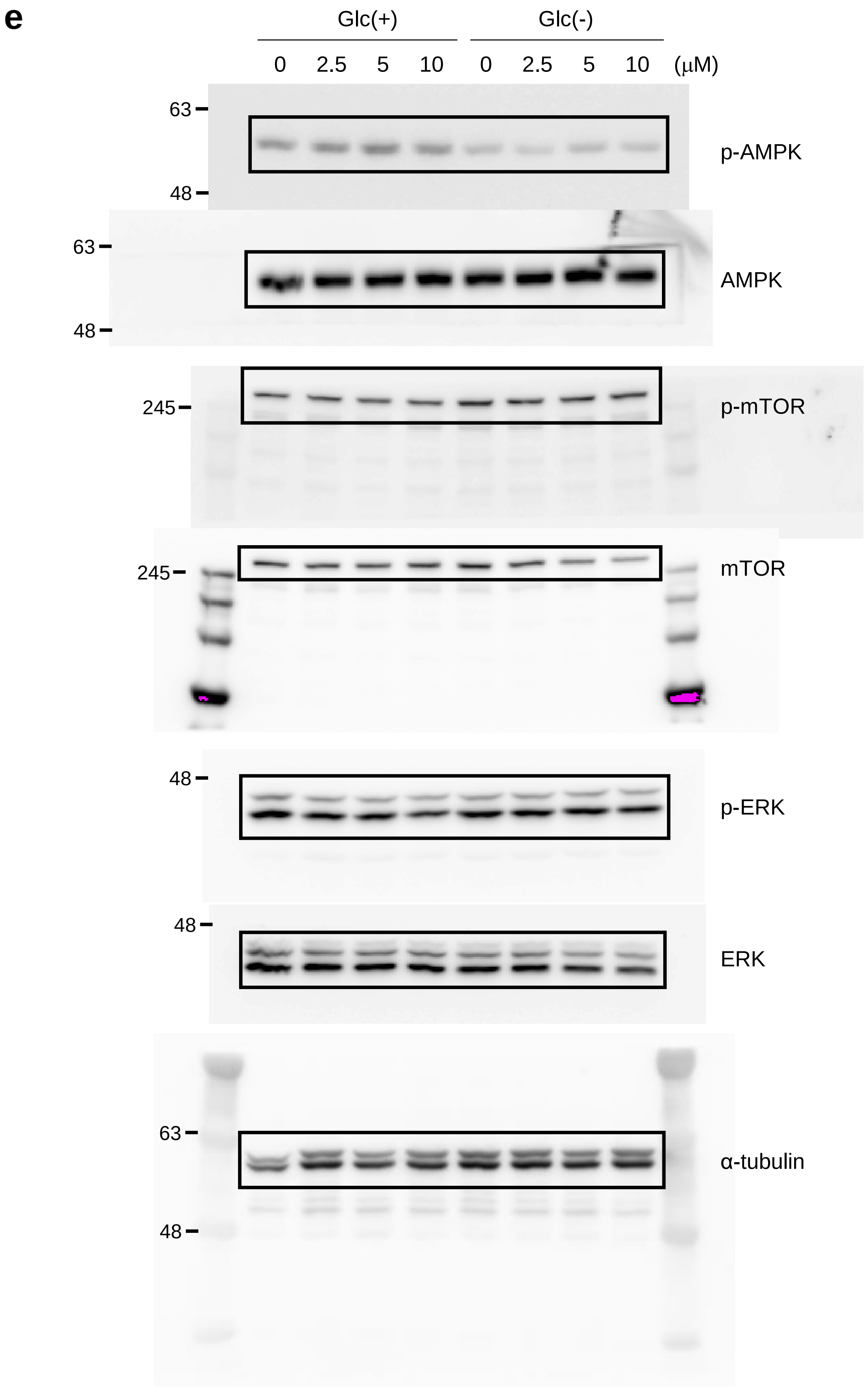
e
Glc(+)
Glc(-)
0
2.5
5
10
0
2.5
5
10
(μM)
63
48
p-AMPK
63
48
AMPK
245
p-mTOR
245
mTOR
48
p-ERK
48
ERK
63
48
α-tubulin
